# Endothelial Snail Regulates Capillary Branching Morphogenesis via Vascular Endothelial Growth Factor Receptor 3 Expression

**DOI:** 10.1371/journal.pgen.1005324

**Published:** 2015-07-06

**Authors:** Jeong Ae Park, Dong Young Kim, Young-Myeong Kim, In-Kyu Lee, Young-Guen Kwon

**Affiliations:** 1 Department of Biochemistry, College of Life Science and Biotechnology, Yonsei University, Seoul, Korea; 2 Vascular System Research Center, Kangwon National University, Kangwon-Do, Korea; 3 Department of Internal Medicine, Kyungpook National University School of Medicine and Leading-edge Research Center for Drug Discovery and Development for Diabetes and Metabolic Disease, Kyungpook National University Medical Center, Daegu, Korea; University of North Carolina, UNITED STATES

## Abstract

Vascular branching morphogenesis is activated and maintained by several signaling pathways. Among them, vascular endothelial growth factor receptor 2 (VEGFR2) signaling is largely presented in arteries, and VEGFR3 signaling is in veins and capillaries. Recent reports have documented that Snail, a well-known epithelial-to-mesenchymal transition protein, is expressed in endothelial cells, where it regulates sprouting angiogenesis and embryonic vascular development. Here, we identified Snail as a regulator of VEGFR3 expression during capillary branching morphogenesis. Snail was dramatically upregulated in sprouting vessels in the developing retinal vasculature, including the leading-edged vessels and vertical sprouting vessels for capillary extension toward the deep retina. Results from *in vitro* functional studies demonstrate that Snail expression colocalized with VEGFR3 and upregulated *VEGFR3* mRNA by directly binding to the *VEGFR3* promoter via cooperating with early growth response protein-1. Snail knockdown in postnatal mice attenuated the formation of the deep capillary plexus, not only by impairing vertical sprouting vessels but also by downregulating VEGFR3 expression. Collectively, these data suggest that the Snail-VEGFR3 axis controls capillary extension, especially in vessels expressing VEGFR2 at low levels.

## Author Summary

Endothelial cells have the intrinsic capacity to shuffle between tip, stalk, and phalanx cells in angiogenic processes. These transitions require the induction or repression of transcripts that are specific for their phenotypes, along with morphological changes. To gain insight into spatiotemporal induction during vascular branching morphogenesis, we used Affymetrix oligonucleotide arrays to screen for Snail. Then, we used stable, small-interfering RNA or the lentivirus-short hairpin RNA system to examine the angiogenic roles of endothelial Snail during retinal capillary morphogenesis. Knockdown of Snail in the developing retinal vasculature impaired deep capillary formation and attenuated vascular endothelial growth factor receptor 3 expression, indicating a functional link between Snail and vascular endothelial growth factor receptor 3. Moreover, we showed vascular endothelial growth factor receptor 3 as a transcriptional target of Snail *in vitro*. In the retinal vasculature, the deep capillary plexus is a unique vessel with only capillary. The deep capillary plays a critical role in retinal development, neuronal survival, and pathological conditions, including ischemic diseases. Our findings provide molecular insights into the role of the Snail-vascular endothelial growth factor receptor 3 axis in capillary formation under pathophysiological conditions.

## Introduction

During vascular morphogenesis, new vessels sprout from existing ones to generate a functional and hierarchical branched network [1]. The retina has widely been used as a model system to investigate the mechanism of vascular morphogenesis [2]. The retinal vasculature is composed of the superficial and deep plexus (S2A Fig) [3]. The superficial vascular plexus is a well-differentiated structure of arteries, veins, and capillaries, whereas the deep vascular plexus is composed of capillaries. At birth, mice have avascular retinas. By the first postnatal day (P1), the vessels emerge at the optic stalk and initially form the superficial vascular plexus, which begins centrally and proceeds peripherally. By P8, the vessels are rapidly remodeled into a hierarchical structure that consists of arteries, veins, and capillaries. Beginning around P7, vertical angiogenic sprouting generates from the mature part of the superficial plexus and penetrates into deep retinal layers. When the vertical vessels reach the inner and outer boundaries of the inner nuclear layer (INL), the vessels turn sideways, sprout, and fuse to establish the deep vascular plexus. Vertically sprouting vessels can sense and respond to attractive and repulsive signals within their immediate microenvironment from the ganglion cell layer (GCL) through the INL to the outer plexus layer (OPL).

In the vasculature, several signaling pathways control endothelial cell (EC) sprouting, migration, and network expansion [1]. Examples of these signaling components are Notch, vascular endothelial growth factor receptor (VEGFR) 2/3, Delta-like ligand 4 (Dll4), and bone morphogenetic proteins (BMPs). Dll4 expression is dynamically regulated and associated with actively growing vessels, but its expression is gradually reduced with the cessation of angiogenic sprouting in the superficial plexus. In mature vessels of mice at P9, Dll4 is expressed in arteries rather than in veins [4]. VEGFR2 and 3 are expressed in the specialized tip cells of actively growing vessels. After the maturation of the superficial plexus, VEGFR2 is largely detected in arteries, and VEGFR3 is expressed in veins and capillaries [5,6]. VEGFR2 triggers multiple downstream signals and consequently stimulates ECs to guide proper angiogenic sprouting vessels, filopodia extension, and network expansion. On the other hand, VEGFR3 has dual activities, where it can promote but also inhibit angiogenesis [7]. VEGFR3 blocks angiogenesis by interfering with VEGFR2 signaling in the superficial plexus. VEGFR3 signaling can also be pro-angiogenic via VEGFC- and extracellular matrix (ECM) component-mediated signals, and it plays an important role in venous angiogenesis and lymphangiogenesis [7–9]. However, the induction mechanism and functional roles of proangiogenic VEGFR3 remain largely unknown.

The Snail family of zinc-finger transcription factors is comprised of Snail1 (Snail), Snail2 (Slug), and Snail3 [10]. Snail is localized to the cytoplasm and nucleus, whereas Slug is localized to the nucleus. Most functions of the Snail family, such as epithelial-to-mesenchymal transition (EMT), survival, cell motility, and cell movement, have been studied in epithelial cells [11,12]. During epithelial branching morphogenesis, epithelial cells induce the expression of the Snail family at the leading edge of growing branches and appear to undergo EMT [13]. Furthermore, in the *Drosophila* trachea, branchless (a fibroblast growth factor ligand) signaling establishes tip/stalk cells and controls the expression of escargot, which is a *Drosophila* homolog of Slug that is involved in branch fusion [14]. Recent studies have demonstrated remarkable similarities between epithelial morphogenesis and angiogenic sprouting with regard to the organization of sprouting cells into the tip and stalk, cell migration, and fusion between tip cells [1].

Accumulating evidence has indicated that the Snail family may participate in vascular branching morphogenesis. The vascular effects of Snail have been revealed in embryos of mice with the epiblast-specific deletion of *Snail* [15]. *Snail* deletion results in the failure to form appropriately interconnected vascular networks. In *Xenopus* vascular development, the ectopic expression of Slug/Twist is sufficient to rescue a Myc knockdown-induced vascular defect [16]. Notably, in extracted lysates from Dll4^+/-^ retinal ECs, Slug is expressed in highly motile tip cells [17]. More recently, Slug has been shown to be associated with sprouting angiogenesis by inducing membrane type 1-matrix metalloproteinase (MT1-MMP) *in vitro* [18]. Parker *et al*. [19] have demonstrated that Snail is detected in the extracts of ECs isolated from invasive breast ductal tumors; however, Snail is undetectable in the normal breast. Although the evidence seems to support a role for the Snail family in the developing vessels and tumor vasculature, the precise expression pattern and cellular function of Snail in vascular morphogenesis remain unclear.

To gain insight into the spatiotemporal induction of global genes during vascular morphogenesis, we used Affymetrix oligonucleotide arrays (GRE accession number GSE12891) to compare their mRNA levels at time points that corresponded to dramatic morphological changes during EC network formation. In this study we showed that Snail was dynamically and predominantly expressed in active vessels. We evaluated the role of Snail on VEGFR3 in capillary branching morphogenesis.

## Results

### Identification of Snail in the network formation *in vitro* and in sprouting vessels in the developing retina

**Fig 1:**
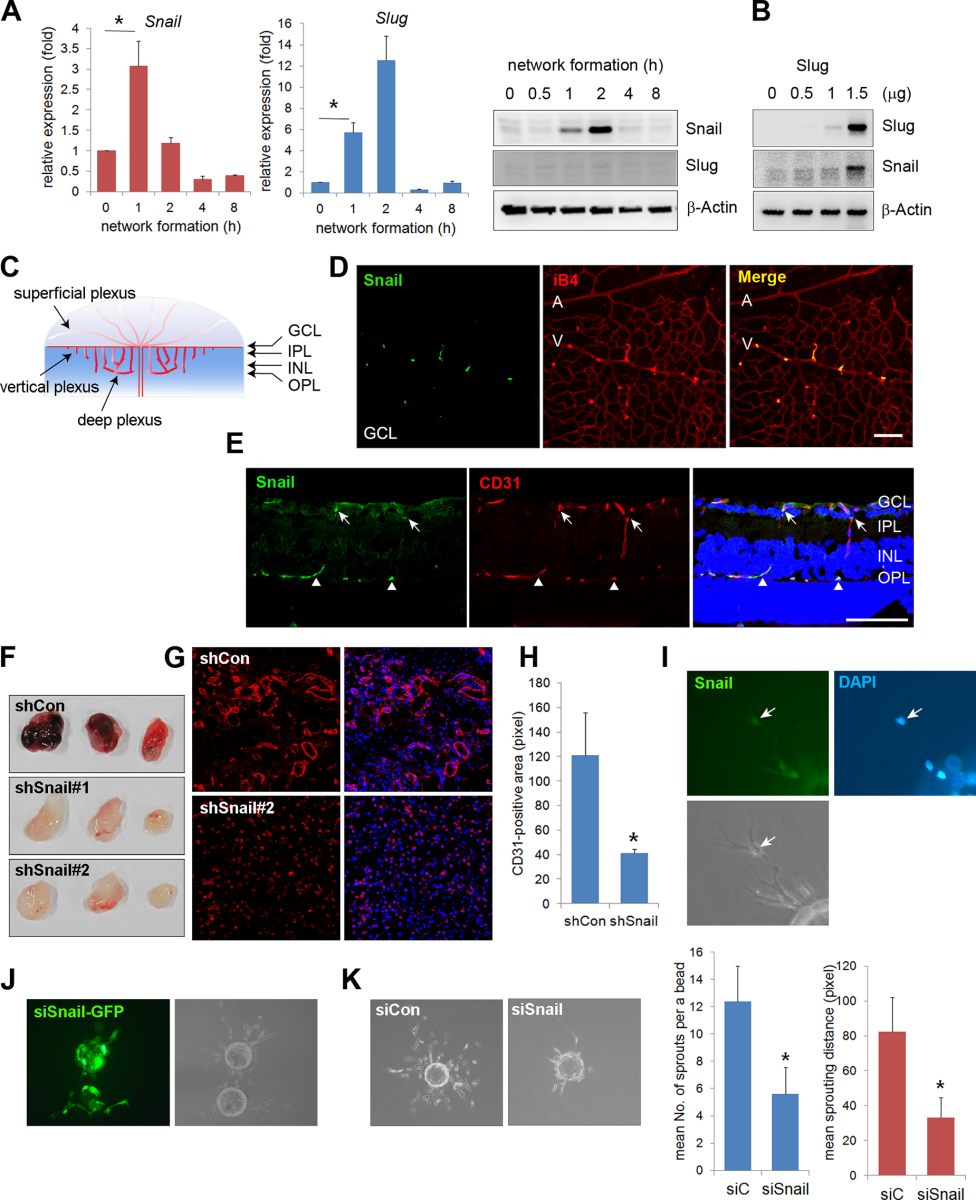
Snail is expressed in sprouting vessels. (A) Quantitative reverse transcription-polymerase chain reaction (RT-PCR) (left and middle) and western blot (right) analyses showing the expression pattern of Snail and Slug during *in vitro* vascular network formation. Human umbilical vein endothelial cells (HUVECs) were placed on Matrigel and analyzed at the indicated time points. *, p<0.001. (B) Western blot analysis showing Slug-mediated Snail induction. Slug was transfected with the indicated doses in HUVECs. On the next day, the cells were lysed, and western blot analysis was performed. (C) Illustration of the developing retinal vessel from the superficial plexus to the deep plexus in mice at postnatal day 11 (P11). The superficial plexus is represented by vessels around the ganglion cell layer (GCL), the vertical vessel includes vessels around the inner plexiform layer (IPL) and inner nuclear layer (INL), and the deep plexus is represented by vessels around the outer plexiform layer (OPL). (D) Confocal images showing Snail immunoreactivity. Whole flat-mount staining analysis was performed in eyeballs at P8. The immunoreactivity of Snail (green) was observed in sprouting vessels from the vein. A, artery; V, vein; iB4, isolectin B4. Bar, 100 μm. (E) Cross-sectional confocal images at P11 showing Snail expression in the descending vessels. Sections were stained with anti-Snail (green) and anti-CD31 (red) antibodies. The immunoreactivity of Snail was detected in the superficial branching region (GCL and IPL; arrows) and the vertical vessels (INL; triangles). Bar, 100 μm. (F) Representative images of Matrigel plugs at 6 days after the subcutaneous injection of Matrigel plugs containing the small hairpin (sh)Lenti Snail virus and vascular endothelial growth factor A (VEGFA; 200 ng/mL) into C57BL/6 mice (n = 6 per group). Two types of shLenti Snail virus (shSnail#1 and shSnail#2) were used. (G) Immunohistochemical analysis showing infiltrating mouse CD31^+^ ECs (red). The Matrigel plug containing the shLenti Snail virus (shSnail#2) recruited mouse ECs but failed to initiate vascular network formation. (H) Quantification of vessel ingrowth by measuring CD31^+^ length (right). *, p<0.01. (I) Snail immunofluorescence in a fibrin gel bead after one day of culture. The cells were stained with anti-Snail antibodies (green). Nuclei were DAPI-positive (blue). (J) Immunofluorescence images of the mixed culture of control siCon and siSnail-GFP- transfected HUVECs. SiSnail was transfected in GFP-overexpressing HUVECs, and siCon was transfected in HUVECs before mixed culture (1:1) on fibrin beads. Most of the siSnail-GFP-transfected cells remained on the beads, whereas siCon-transfected cells sprouted to the fibrin gel. siSnail, small-interfering RNA targeting Snail. (K) Fibrin bead assay showing representative images by siCon- and siSnail-transfected HUVECs (left). Sprouting numbers per bead or sprouting lengths from one bead were calculated to quantify endothelial sprouting (right). *, p<0.01.

Affymetrix oligonucleotide arrays (GRE accession number GSE12891) were used to compare the mRNA levels of global genes at time points that corresponded to dramatic morphological changes during vascular morphogenesis. Specifically, we looked for genes that were altered during EC network formation, because they may influence endothelial morphological changes in response to cell-cell and cell-ECM interactions (S1 Fig). *Snail* and *Slug* expression levels were dramatically increased in those processes. Quantitative reverse transcription-polymerase chain reaction (qRT-PCR) and western blot analyses confirmed that Snail mRNA and protein levels were dramatically increased at 1 and 2 h when the behavior of ECs was robust (Figs 1A and S1A). At 4 h when vascular network formation was complete, Snail expression disappeared. Although *Slug* mRNA expression dramatically increased, Slug protein levels could not be detected, thus suggesting that Slug protein is highly unstable during vascular network formation (Fig 1A, middle and right). Furthermore, we found that ectopic expression of Slug in human umbilical vein ECs (HUVECs) dramatically increased Snail, which suggests that Slug could be upstream of Snail (Fig 1B). Similar to our finding, Slug has been reported to be indirectly involved in epithelial branching via Snail upregulation [13]. The differential function between Snail and Slug has been suggested, such that Slug is predominantly effective in cell survival, whereas Snail is involved in invasive and migrating events. Hence, we focused on the role of Snail in the angiogenic process, although Snail and Slug appear to play roles in vascular morphogenesis.

We next investigated whether Snail was involved in vascular development *in vivo*. The retinal vasculature is composed of the superficial plexus and deep plexus (Figs 1C and S2A) [20]. Whole flat-mount analysis showed that the immunoreactivity of Snail was found in active vessels at P5 (S2B Fig). Z-stack analysis indicated that Snail was located in the nuclei of sprouting cells, as shown in the x-z axis and y-z axis (S2C Fig). At P8, Snail was detected in sprouting vessels from the vein (Fig 1D). At P11, the deep plexus was observed, and Snail was detected in the vertical vessel of the cross-section and in whole flat-mount retinas (Figs 1E and S2D). In particular, Snail immunoreactivity was detected in the IPL and INL where vessels are sprouting and branching (S2D and S2E Fig). However, Snail expression was not detected after completion of the deep plexus (S2D Fig, OPL). These results demonstrate that Snail was expressed in the sprouting vessels prior to vascular plexus formation.

### Knockdown of Snail attenuates vascular sprouts

The role of Snail in vascular sprouting was examined in HUVECs using the Matrigel plug assay. Matrigel plugs containing the small hairpin (sh) Lenti Snail virus (shSnail) attenuated vascular formation compared with the shLenti control virus (shCon) (Fig 1F). Immunohistochemical analysis indicated that mouse ECs infiltrated into Matrigel plugs containing shSnail, but the vessel ingrowth abilities of ECs displayed deficits (Fig 1G and 1H). In the fibrin gel setting, Snail was detected in sprouting ECs (Fig 1I). In mixed culture, Snail small-interfering RNA (siSnail)-transfected HUVECs failed to sprout and migrate toward the fibrin-gel matrix, and the ECs remained on the beads, whereas control siRNA (siCon)-transfected HUVECs migrated and sprouted (Fig 1J). Knockdown of Snail attenuated the ability of ECs to sprout from the bead (Fig 1K). Furthermore, we embedded individual ECs inside a three-dimensional fibrin gel and assessed the sprouting ability of the cells (S3 Fig). The sprouts from siSnail-transfected ECs exhibited a dead-end morphology, whereas those from siCon-transfected ECs sharply extended within the fibrin gel (S3A Fig, arrows). SiSnail-transfected ECs were also less able to form tubes (S3A Fig). In contrast, ectopic Snail increased EC sprouting and branching (S3B Fig, arrow head). Furthermore, ectopic expression of Snail (6SA), an unleashed form from GSK3β-proteosomal degradation, enhanced the ability of EC sprouting (S3B Fig). Therefore, the data suggest that Snail is essential for the initiation and induction of vascular sprouting and branching.

### Snail is upregulated by ECM-mediated signals

**Fig 2:**
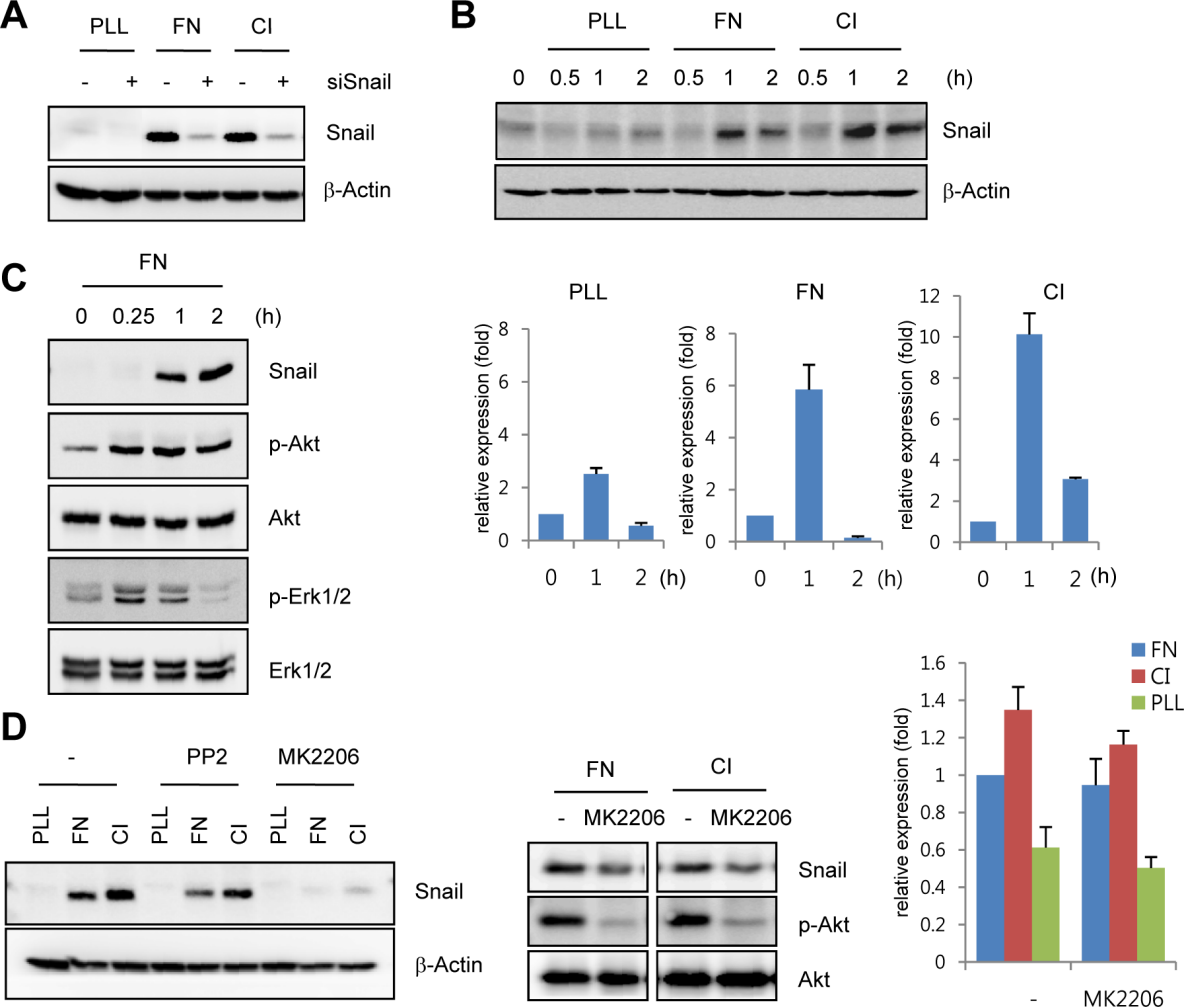
Snail is upregulated under extracellular matrix (ECM)-mediated signals. (A) Western blot analysis showing Snail expression on immobilized ECM. After HUVECs were transfected with siCon or siSnail, the transfectants were reseeded and cultured on PLL (20 μg/mL)-, FN (20 μg/mL)-, or CI (20 μg/mL)-coated culture dishes for 2 h. PLL, poly-L-lysine; FN, fibronectin; CI, collagen type I. (B) Time-course expression pattern of Snail on immobilized ECM. Confluent HUVECs were reseeded and cultured on PLL-, FN- or CI-coated dishes for the indicated time points. Snail expression was evaluated by western blot (upper) and quantitative RT-PCR (lower) analyses. (C) Western blot analysis showing the induction of phosphorylated Akt (p-Akt) and phosphorylated extracellular-regulated kinase 1/2 (p-Erk1/2) in HUVECs that were cultured on FN-coated dishes. (D) Snail expression on immobilized ECM after MK2206 treatment. Confluent HUVECs or human retinal endothelial cells (HRECs) were pre-exposed to 10 μM PP2 (a Src kinase inhibitor) or 1 μg/mL MK2206 (an allosteric Akt inhibitor) for 1 h, followed by reseeding and culture on PLL-, FN-, or CI-coated dishes for 2 h (western blot) or 1 h (quantitative RT-PCR).

The developing retinal vasculature into the deep retinal layer is influenced by ECM-mediated integrin signals and retinal neuron-induced hypoxic signals [3,21]. On the basis of the finding that Snail immunoreactivity was detected in the invading vessels into surrounding matrix microenvironment, we investigated the mechanism by which ECM could regulate Snail expression. Fibronectin and collagen type I are major ECM components that are involved in angiogenesis [22]. Exposure of HUVECs to these ECM components dramatically induced Snail protein and mRNA expression (Fig 2A and 2B). In comparison, exposure of HUVECs to poly-L-lysine (PLL), a non-specific adhesion facilitator, only slightly induced Snail protein and mRNA expression. In normal, cultured ECs, Snail protein is unstable and can only be detected in the presence of proteosome inhibitors [10]. Several studies examining the stability of Snail protein have shown that Snail is rapidly degraded via the glycogen synthase kinase (GSK) 3β-dependent proteosomal system in epithelial cells. Activated Akt can phosphorylate GSK3β, and this process stabilizes Snail by releasing it from the GSK3β system [23]. We found that exposure of HUVECs to the ECM induced Akt phosphorylation (Fig 2C). Thus we examined whether the maintenance of Snail protein on ECM component was due to Akt activity. Pretreatment with MK2206 (an allosteric Akt inhibitor) attenuated fibronectin- and collagen type I-mediated Snail induction in protein level (Fig 2D, left and middle). In contrast, mRNA level of Snail showed slight decrease (Fig 2D, right). Although further experiments are required to better understand the transcriptional regulation of *Snail* by ECM signaling, these results suggest that ECM-induced Snail protein in ECs was stabilized by Akt signals, which prevented Snail from GSK3β-proteosomal degradation.

### Snail upregulates VEGFR3 expression via cooperation with Egr-1

On the basis of our finding that Snail was dominantly expressed in the sprouting vessels and regulated by ECM signals, we investigated whether Snail influenced the expression of EC sprouting-related genes, including VEGFRs and Neuropilin (NRP). In particular, we have focused on VEGFR3, because VEGFR3 is known to interact with ECM and ECM ligands, including fibronectin and integrin α5β1 [9,24]. Furthermore, VEGFR3 is highly expressed in leading-edged ECs that undergo sprouting and migration but is weakly and rarely expressed in phalanx ECs and quiescent ECs [1]. Thus, VEGFR3 needs to be induced for resting ECs to initiate angiogenesis. Most studies on VEGFR3 expression have focused on lymphatic ECs, and VEGFR3 is induced by the formation of prospero homeobox protein 1 (Prox1)-COUP transcription factor 2 (CoupTFII), Prox1-nuclear factor-κB, or Prox1-Ets complexes [25,26]. In blood ECs, the binding of Notch to the *VEGFR3* promoter can induce *VEGFR3* mRNA [27]. However, the induction mechanism of VEGFR3 in angiogenically active blood ECs is largely unknown.

**Fig 3:**
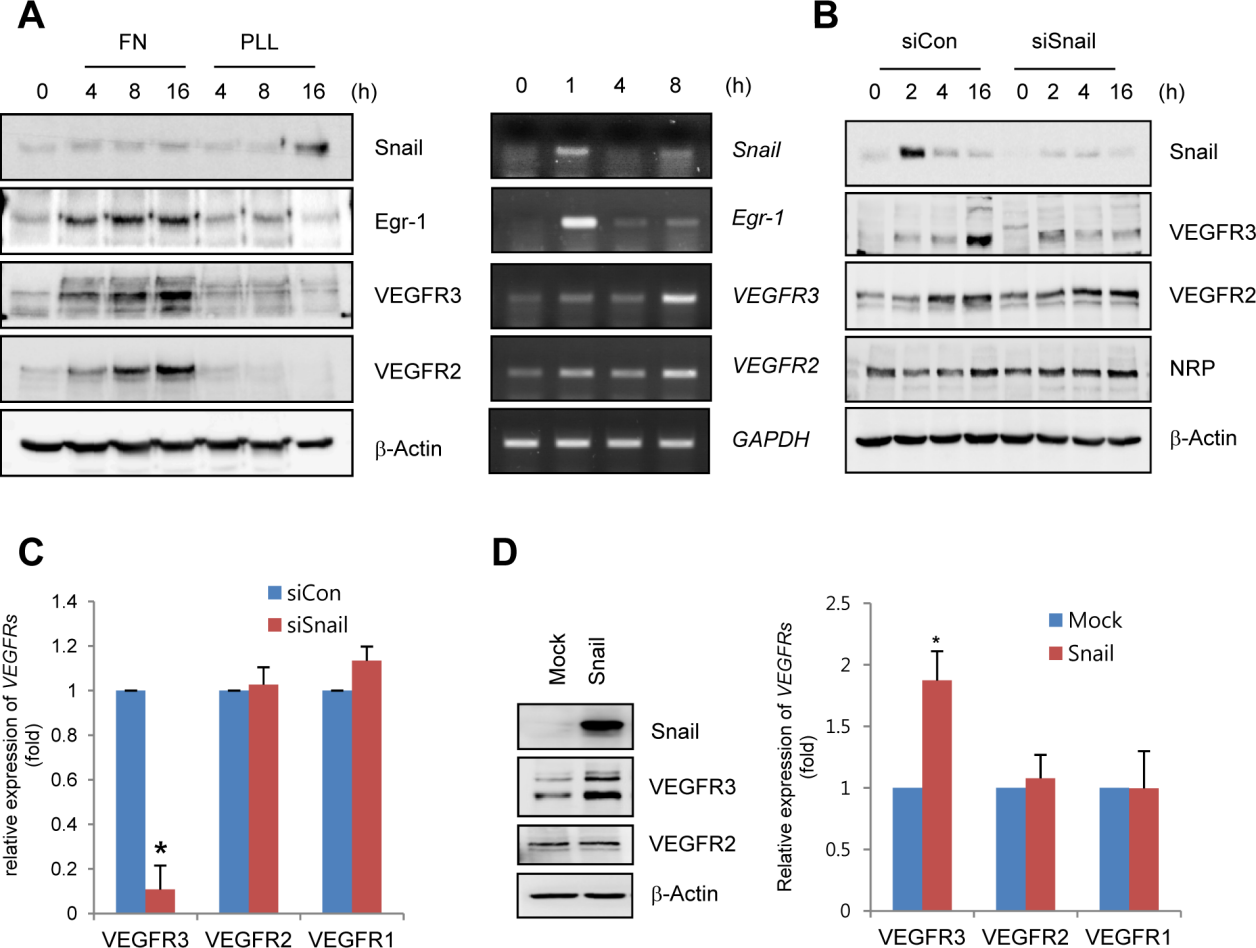
Snail upregulates VEGF receptor 3 (VEGFR3). (A) Western blot and RT-PCR analyses showing Snail, early growth response protein-1 (Egr-1), VEGF receptor 3 (VEGFR3), and VEGFR2 expression. HRECs were seeded at a density of 2–2.5×10^4^ cells/cm^2^ on FN- (for western blot and RT-PCR) or PLL (for western blot)-coated dishes and cultured for the indicated time points. (B) Western blot analysis showing the effect of Snail knockdown on VEGFR3. HRECs were reseeded after transfections with siCon or siSnail on FN-coated dishes, and cultured for the indicated time. (C) Quantitative RT-PCR analysis show^i^ng the effect of Snail knockdown on VEGFR3 expression. SiSnail-transfected ECs were reseeded and cultured on FN-coated dishes for 8 h. *, p<0.01. (D) Western blot and quantitative RT-PCR analyses showing the effect of Snail overexpression on VEGFR3. HUVECs were transfected with Snail. On the next day, the medium was changed, and the transfected cells were cultured for 8 h (quantitative RT-PCR; right) or 16 h (western blot; left). *, p<0.01.

To determine whether ECM could induce the expression of VEGFRs in ECs, human retinal endothelial cells (HRECs) and HUVECs were exposed to fibronectin. Fibronectin, but not PLL, dramatically increased VEGFR3 mRNA and protein expression (Figs 3A and S4A). Interestingly, Snail knockdown with siRNA reversed ECM-mediated VEGFR3 upregulation at the protein and mRNA levels but showed no effect on VEGFR2 and NRP (Fig 3B and 3C). The increase in VEGFR3 was confirmed by the ectopic expression of Snail (Fig 3D).

**Fig 4:**
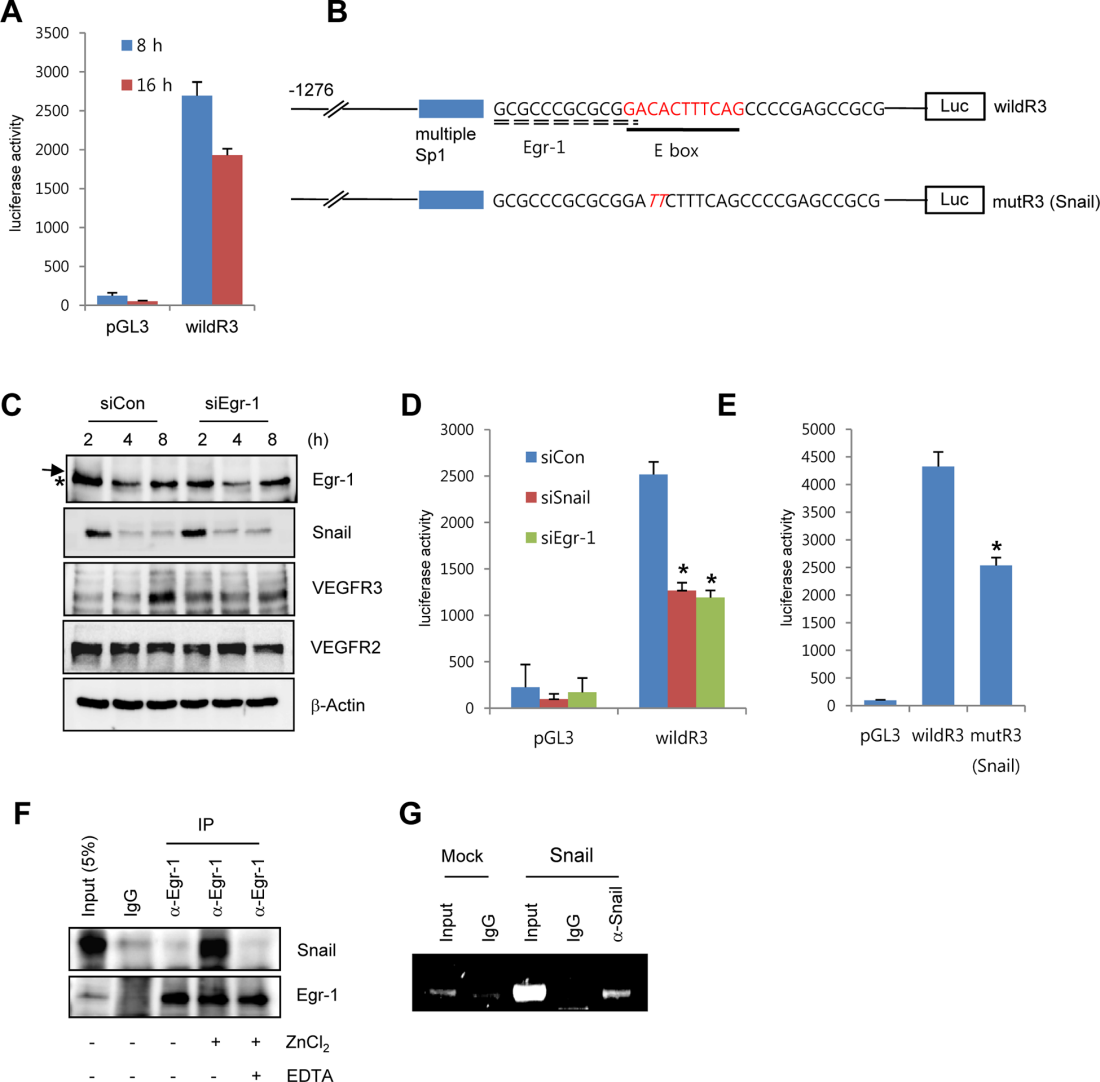
Snail upregulates VEGFR3 transcripts via cooperating with Egr-1. (A) *VEGFR3* promoter activity after the exposure of HRECs to immobilized FN. HRECs were transfected with the human *VEGFR3* promoter_luciferase (hVEGFR3_Luc) reporter (wildR3) and then reseeded at a density of 2–2.5×10^4^ cells/cm^2^ on FN-coated dishes. (B) Schematic illustration of the location of putative Snail and the Egr-1-binding site in the human *VEGFR3* promoter. WildR3, human *VEGFR3* promoter_luciferase (hVEGFR3_Luc) reporter; mutR3(Snail), mutation in the putative E-box. Broken line, Egr-1-binding elements; thick line, putative E-box; Luc, luciferase. (C) Western blot analysis showing the effect of Egr-1 knockdown on VEGFR3. HRECs were reseeded after transfections with siCon or siEgr-1 on FN-coated dishes. Arrow, an Egr-1 band; *, a non-specific band. (D) *VEGFR3* promoter activity after the knockdown of Snail or Egr-1. HRECs were co-transfected with the indicated siRNA and the wildR3 reporter and then reseeded and cultured on FN-coated dishes for 16 h. (E) Mutant *VEGFR3* promoter activity. HRECs were transfected with the indicated wildR3 and mutR3 (Snail) reporters and then reseeded on FN-coated dishes for 16 h. (F) Immunoprecipitation assay demonstrating the complex association between Snail and Egr-1. HRECs were seeded on FN-coated dishes. After 2 h, the cell lysates were immunoprecipitated (IP) with immunoglobulin G (IgG) or anti-Egr-1 antibody (α-Egr-1). (G) Chromatin immunoprecipitation analysis of the *VEGFR3* promoter in HUVECs. HUVECs were transfected with flag-Snail (Snail) and immunoprecipitated using anti-Snail antibodies (α-Snail). PCR was performed to detect the *VEGFR3* promoter region containing the putative E box.

To explore whether Snail mediated VEGFR3 via the enhancement of *VEGFR3* promoter activity, we employed the luciferase reporter system. Exposure of ECs to ECM components enhanced *VEGFR3* promoter activity (Fig 4A). *VEGFR3* promoter activity was downregulated and upregulated by Snail knockdown and ectopic Snail, respectively (Figs 4D, S4B and S4C). Because Notch activates *VEGFR3* promoter activity [27], we examined whether the ECM-mediated increase in VEGFR3 was Notch dependent. Notch siRNA (siNotch) transfection slightly downregulated *VEGFR3* promoter activity. A similar effect was observed with DAPT, which is an inhibitor of the γ-secretase and Notch response (S4D Fig). Therefore, the intracellular domain of Notch is unlikely to be a transcriptional regulator of VEGFR3 under the influence of ECM in our system. The Snail family is known to act as a transcriptional repressor for tight junction genes, polarity-related genes, and cell cycle regulators by directly binding to their conserved E-box element [10]. Nonetheless, many genes are also upregulated by the Snail family, which suggests that it functions as a transcriptional activator. Several reports indicated that Snail interacts and cooperates with the Egr-1/Sp1 complex to enhance the promoter activity of its target genes, and Egr-1 is implicated in several vascular disease states and fibroblast growth factor 2-mediated angiogenesis [28–30]. By screening the TRANSFAC MATRIX TABLE, we found that the promoter region of human *VEGFR3* contained multiple conserved Sp1-binding sites, a nearby conserved Egr-binding element, and a putative E-box element located within approximately 200 bp upstream from the initiation of *VEGFR3* mRNA (Fig 4B). Exposure of ECs to fibronectin induced Egr-1, Snail, and VEGFR3 (Figs 3A and 4C). Knockdown of Egr-1 decreased VEGFR3 protein expression and *VEGFR3* promoter activity, suggesting the involvement of Egr-1 in *VEGFR3* transcription (Figs 4C, 4D, and S4B).

To examine direct involvement of Snail in *VEGFR3* promoter activity, we performed the mutagenesis of putative E-box elements (Fig 4B). Site-directed mutagenesis of the *VEGFR3* promoter region significantly reduced Snail-induced *VEGFR3* promoter activity, thus demonstrating the requirement of Snail for *VEGFR3* promoter activity (Fig 4E). To determine whether the intimate binding region of the E-box and Egr-1 in the *VEGFR3* promoter could lead to the interaction between Snail and Egr-1, we exposed HRECs to fibronectin for 2 h to induce Snail and Egr-1 and performed the immunoprecipitation assay. Because Snail is a zinc-finger transcription factor, we added the zinc ion to HREC lysates. Incubation of EC lysates with anti-Egr-1 revealed the interaction between Egr-1 and Snail (Fig 4F). Co-treatment with ethylenediaminetetraacetic acid (EDTA), a zinc chelator, inhibited the binding, which suggests that the interaction of Egr-1 with Snail is specific and is apparently related to its transcriptional activity (Fig 4F). To examine the direct binding of Snail to the promoter region, we performed chromatin immunoprecipitation (ChIP) analysis in Snail-overexpressing HUVECs. The *VEGFR3* promoter region containing Snail and Egr-1-binding sites was co-immunoprecipitated with anti-Snail antibodies (Fig 4G).

These results demonstrate that Snail and Egr-1 were induced and stabilized under ECM signals. Subsequently, Snail bound to the *VEGFR3* promoter through Egr-1 cooperation, thus leading to the transcriptional activation of *VEGFR3* in ECs.

### Vertical vessels strongly express VEGFR3, but not VEGFR2, in the deep retinal vasculature

Growing venous and capillary vessels have dominant VEGFR3 expression under physiological and pathological conditions, whereas VEGFR2 and Dll4 are strongly expressed in arterial vessels [6]. In the developing retina, the deep vascular plexus is a unique vessel network with capillary vascular plexus. Combined these reports, we assumed VEGFR3 expression in the deep capillary plexus and probed for the expression of VEGFR3 in postnatal retinal angiogenesis. Prior to the experiments, we validated the anti-VEGFR3 antibody that was used in this study by whole-mount cornea staining (S5A–S5C Fig). The immunoreactivity of VEGFR3 colocalized with those of isolectin B4 (iB4; a blood vessel marker) and lymphatic vessel endothelial receptor (LYVE; a lymphatic vessel marker) in the developing cornea, which indicates that the antibody is suitable for detecting VEGFR3.

**Fig 5:**
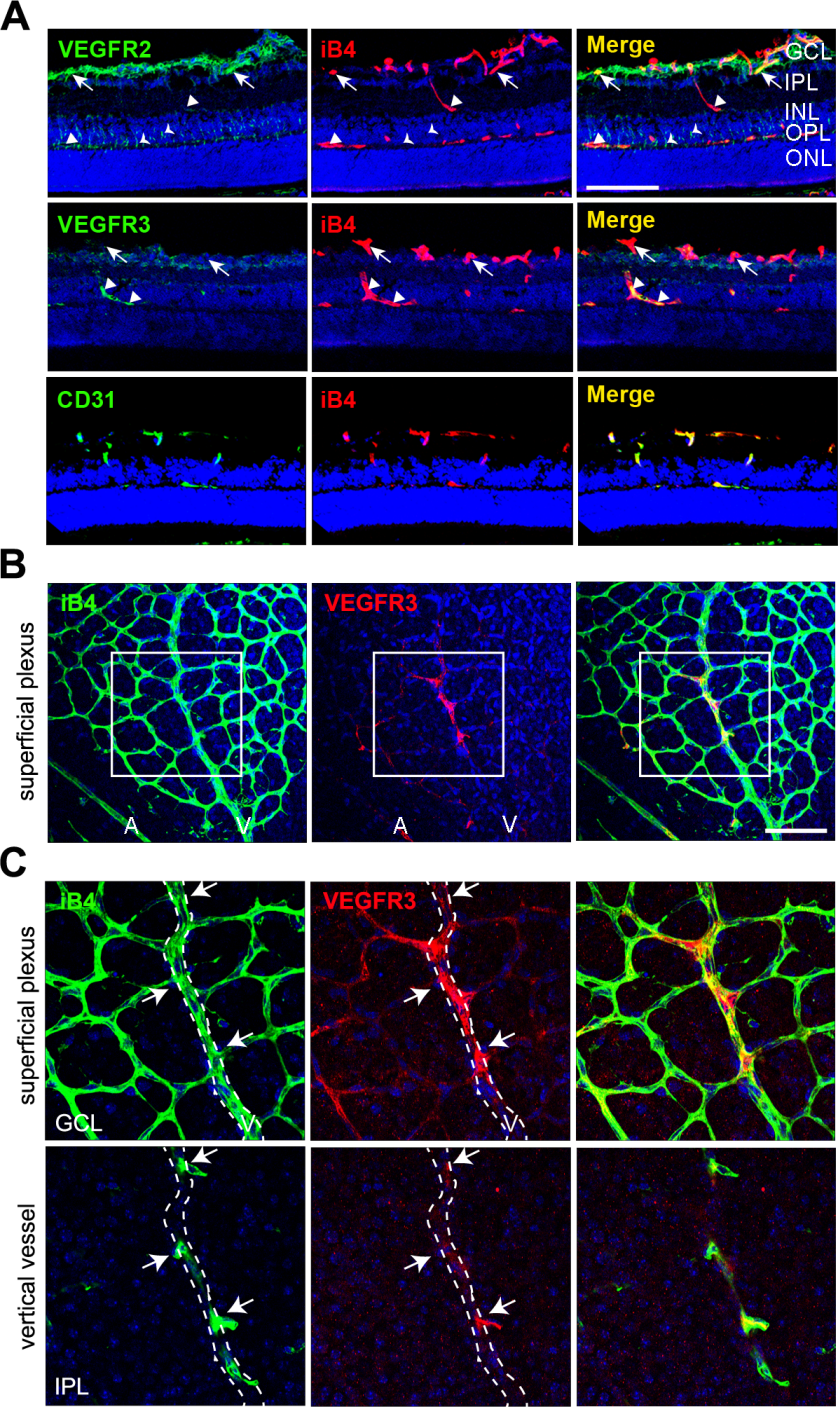
Vertically sprouting vessels have strong VEGFR3, but weak VEGFR2, expression in the developing retinal vasculature. (A) Cross-sectional confocal images showing the differential expression pattern of VEGFR2 and VEGFR3 in P11 mice. The immunoreactivity of VEGFR3 was strongly detected in the vertical vessels (IPL and INL; triangles) and deep plexus (OPL, triangles). In contrast, strong immunoreactivity of VEGFR2 was detected in the superficial plexus (GCL, arrows) and neurons (arrow heads). Nuclei were DAPI positive (blue). ONL, outer nuclear layer. Bar, 100 μm. (**B** and **C**) Confocal images of VEGFR3 staining in the superficial plexus at P8. Eyeballs from P8 mice were applied to whole flat-mount staining of iB4 and VEGFR3. The region in the box (B) is magnified in **C** (upper). The region of the vertical vessel was taken below the superficial plexus. (C, lower) The immunoreactivity of VEGFR3 was detected in sprouting vessels from the vein (arrows). Broken lines correspond to the position of vein that appeared in the superficial plexus. A, artery; V, vein. Nuclei were DAPI positive (blue). Bar, 100 μm.

At P11, the superficial plexus had been fully formed, and it vertically extended toward the deep retina. We determined that the immunoreactivity of VEGFR2 was strong in the GCL of retinas (Fig 5A, upper panel, arrows). In contrast, the immunoreactivity of VEGFR3 was weak in the same area (Fig 5A, middle, arrows). Interestingly, VEGFR3 was strongly detected in vertically invading capillaries toward the deep retina (Fig 5A, middle panel, triangles). Serial z-axis analysis showed that VEGFR3 was highly expressed in deep capillary vessels and migrating and sprouting ECs (S6 Fig). VEGFR2 was barely detected in the vertical vessels, but it appeared to be expressed in neuronal cells (Fig 5A, upper panel, triangles and arrow heads).

Furthermore, we probed for the expression of VEGFR3 in sprouting vessels from venous vessels of the superficial plexus in the P8 retina and found that some venous ECs and sprouting ECs that invaded toward the deep retina showed prominent immunoreactivity of VEGFR3 (Fig 5B and 5C, arrows). The results suggest that VEGFR3 and VEGFR2 are differentially expressed in angiogenic vessels. VEGFR3 was strongly induced by sprouting angiogenic cells toward the deep retina to undergo capillary extension and formation, whereas VEGFR2 was strongly expressed in vessels in the superficial plexus.

### Snail regulates venous sprouting and deep plexus formation *in vivo*

**Fig 6:**
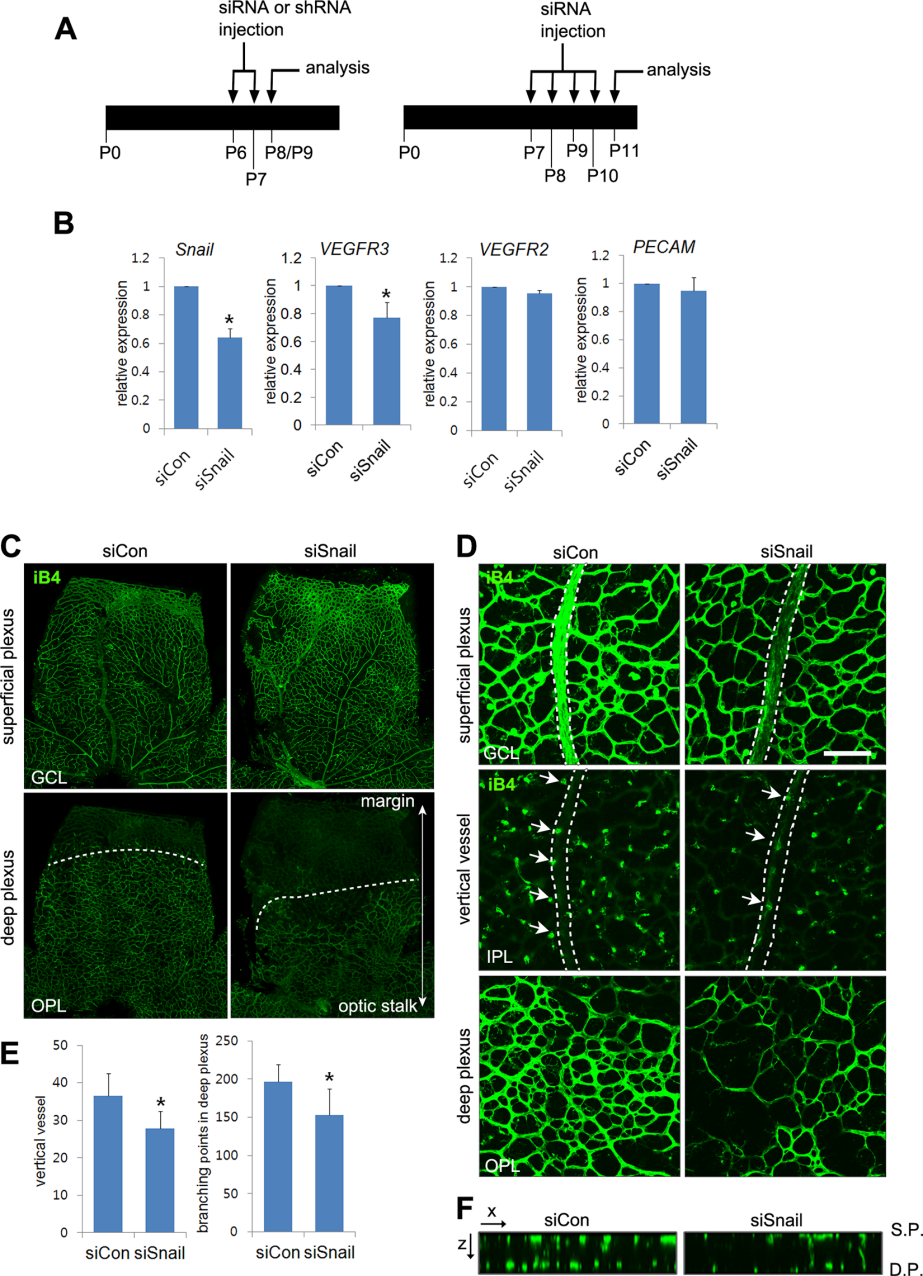
Snail knockdown attenuates retinal vessel sprouting and deep capillary plexus formation. (A) Illustration of the siRNA or shRNA injection strategy in mice. Mice were consecutively and intraperitoneally injected from P6 to P7 or from P7 to P10 and then sacrificed at P8-P9 (P8/P9) or P11, respectively. (B) Quantitative RT-PCR demonstrating Snail knockdown at P11 in siSnail-injected mice. (C) Confocal images of iB4 staining in the superficial plexus and deep plexus. SiSnail or siCon injection was performed, as described in **A**. Whole flat-mount retinas were stained with iB4 at P11. Confocal images were taken in the superficial plexus and then taken in the deep plexus below the superficial plexus by moving the z axis of the confocal microscopic field. The formation of the deep plexus was decreased by Snail knockdown. (D) Representative confocal images of iB4 staining at P11 in siCon- and siSnail-injected mice. SiSnail or siCon injection performed, as described in **A**. Broken lines indicate the position of veins in the superficial plexus. Arrows indicate sprouting vertical vessels from veins in the superficial plexus. Bar, 100 μm. (E) Quantification of vertical vessels and branching points in the deep plexus at P11. *, p<0.05. (F) Confocal images were collected in 1-μm z-stacks in the xz axis at P11 in siCon- and siSnail-injected mice. S.P., the superficial plexus; D.P., the deep plexus.

To examine the role of Snail in the formation of vertical branching and deep capillary plexus, stable Snail siRNA (siSnail) was daily injected into mice from P7 to P10 or from P6 to P8 intraperitoneally (Fig 6A). The efficacy of the siSnail was validated by quantitative RT-PCR at P11 and whole flat-mount analyses at P9 (Figs 6B and S7A). Moreover knockdown of Snail significantly downregulated VEGFR3 expression in whole retinal lysates (Fig 6B). Whole flat-mount analysis showed that the deep plexus was formed from the optic stalk to the retinal margin at P11 (Fig 6C). Snail knockdown impaired the formation of the deep plexus (Figs 6C and S7A, OPL). The distance of the vasculature from the optic stalk to the margin was decreased in siSnail mice. Furthermore, the vertical vessels from the superficial plexus were decreased (Fig 6D, IPL; S7A Fig, GCL). The numbers of vertical vessels that sprouted from the vein were reduced in siSnail retinas and vessel branch points in the deep plexus were also reduced (Figs 6D, 6E, and S7A, IPL and OPL). Confocal z-stack analysis showed the attenuation of vertical vessels in siSnail retinas, compared to siCon retinas (Fig 6F). To avoid the off-target effects of stable siSnail, we utilized the shSnail system, as described in Fig 1F–1H. After intraperitoneal treatments with shShail, whole flat-mount studies showed the reduction in vertical sprouting from the superficial plexus (Figs 6A and S7B). These data demonstrate that Snail played a crucial role in venous vertical sprouting and in the formation of the deep capillary plexus.

### Snail promotes venous sprouting and the formation of the deep capillary network via *VEGFR3* expression

**Fig 7:**
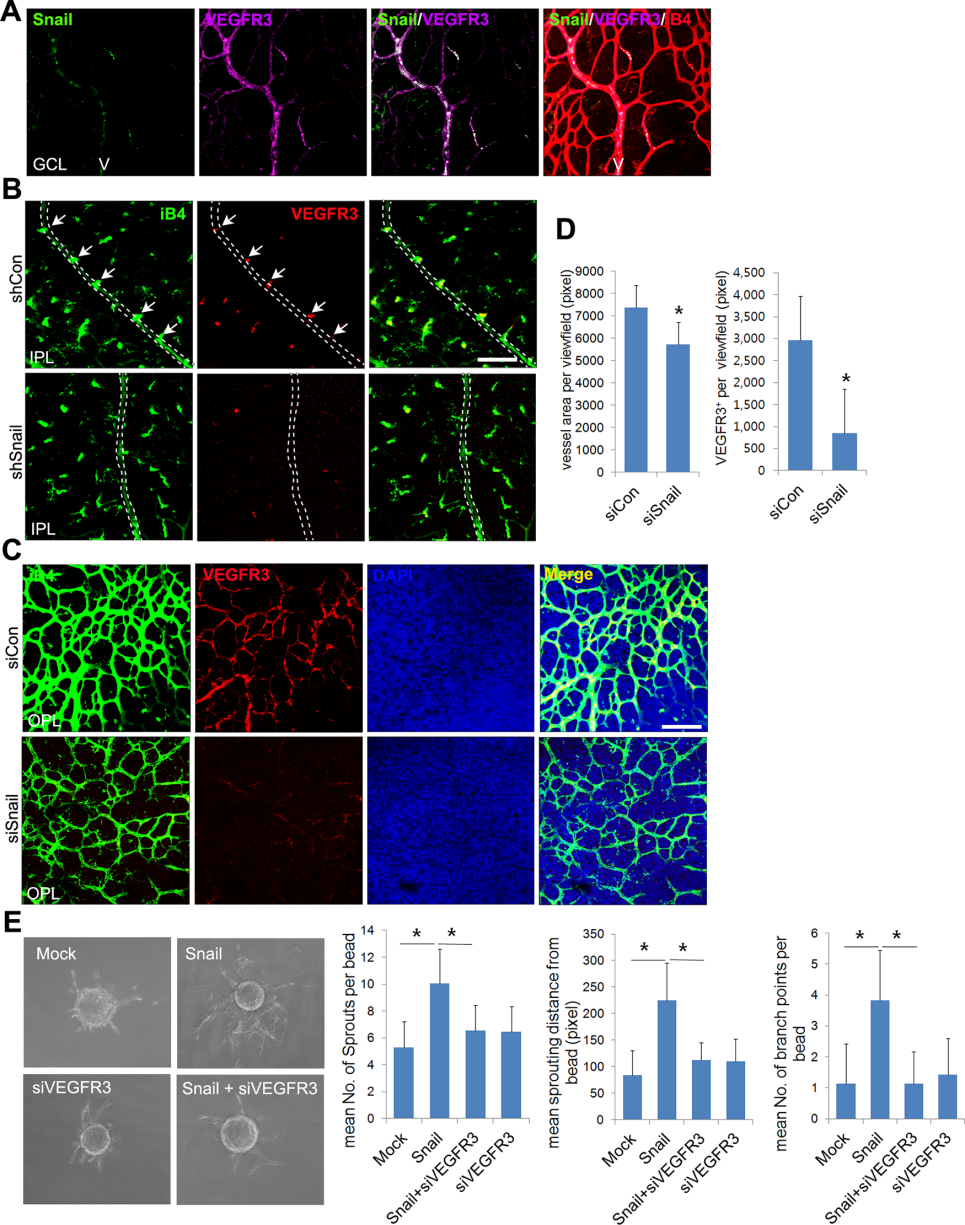
Snail knockdown attenuates VEGFR3 expression in the vertical vessel and the deep plexus. (A) Whole flat-mount images showing the colocalization of Snail and VEGFR3. The immunoreactivity of Snail (green) was observed in the sprouting vessel from the vein in P8 retinal vessels. VEGFR3 immunoreactivity (magenta) was also found in the vein. V, vein. (B) Confocal images of iB4 combined with VEGFR3 staining in shCon or shSnail lentivirus-infected retinas at P8. Mice were consecutively injected intraperitoneally with the shCon or shSnail lentivirus at P6 and P7, as described in Fig 6A. The shSnail lentivirus was the same virus that was described in Fig 1F (shSnail#2). Images of vertical vessels from superficial plexus were taken. Arrows indicate sprouting vessels from veins. Broken line indicates the position of veins in the superficial plexus. Bar, 100 μm. (C) Confocal images of iB4 combined with VEGFR3 staining in the region of the deep capillary plexus in siCon- or siSnail-injected mice at P11. SiRNA injections were performed, as described in Fig 6A. Cell nuclei were stained with DAPI (blue). The immunoreactivity of iB4 and VEGFR3 was weaker in siSnail-injected mice than in siCon mice. Bar, 100 μm. (D) Quantification of total vessel area and VEGFR3-positive regions in the deep plexus at P11. Over six fields were analyzed. *, p<0.01. (E) Fibrin bead assay. HRECs were transfected with mock and Snail in a combination with VEGFR3 siRNA (siVEGFR3). Representative spheroids are shown for each condition (left). Sprouting numbers per bead, sprouting lengths from each bead, and branch numbers were calculated to quantify endothelial sprouting (right). *, p<0.01.

Whole flat-mount staining was performed to assess whether Snail colocalized with VEGFR3 in sprouting vessels (Fig 7A). Expression of VEGFR3 and Snail was high in veins of the GCL at P8, demonstrating that their colocalization could be related to venous sprouting and extension. We thus investigated whether Snail knockdown-induced sprouting defects could be related to VEGFR3 expression *in vivo*. Knockdown of Snail by shSnail injection at consistent intervals reduced both VEGFR3 immunoreactivity and sprouting in the vein at P8 (Figs 6A and 7B). Results from confocal z-axis analysis demonstrate that Snail knockdown reduced the intensity of iB4 immunoreactivity and VEGFR3 expression in retinas at P11 (Figs 7C, 7D and S8).

To investigate whether VEGFR3 triggered the formation of the retinal deep vasculature, we used MAZ51 to block VEGFR3 receptor kinase activity. Treatments with MAZ51 at P4 and P5 significantly reduced the vasculature, radial length, and sprouts at P6 (S9A–S9C Fig). However, MAZ51 treatment from P7 to P10 did not impair the retinal deep vasculature (S9D Fig). The total vessel area and vascular density of MAZ51-treated retinas were not different from that of vehicle-treated retinas (S9E and S9F Fig). These data indicate that the retinal deep vasculature was not dependent on VEGFR3 receptor kinase activity. Similar results have been reported by others [9,31]. They showed that collagen type I-induced VEGFR3 phosphorylation is not blocked by MAZ51 *in vitro* [9]. However, the superficial plexus is inhibited by MAZ51 treatment *in vivo* [31]. Therefore, we speculated that VEGFR3 activation might be different between the superficial and deep vascular plexus in the retina. VEGFR3 has been shown to bind to integrin upon fibronectin exposure and activate downstream signals [7,9]. We examined whether there was a relationship between VEGFR3 and integrins in the formation of the deep vasculature. Whole flat-mount staining and z-stack analysis revealed that venous sprouting vessels showed strong CD29 (integrin β1) immunoreactivity in retinal vessels at P8 (S9G and S9H Fig). The immunoreactivities of VEGFR3 colocalized with those of CD29 in sprouts that extended from the vein (S9I Fig). Next, we asked whether integrin-mediated VEGFR3 activation could induce deep plexus development. *In vitro* experiments have demonstrated that CD29 and c-Src can form a complex with VEGFR3 by integrin engagement to the ECM [9]. They demonstrate that CD29 recruits c-Src, which then phosphorylates VEGFR3. This phosphorylation of VEGFR3 by c-Src is suggested to be distinct from that by VEGFR3 receptor kinase activity [9]. Furthermore, they showed that the exposure of ECs to ECM induces the phosphorylation of VEGFR3 by c-Src, and this process was blocked by PP2, which is a Src family inhibitor. To further investigate the role of the Src family in the development of the deep vasculature, we administered intraperitoneal injections of PP2. PP2 attenuated the formation of the deep vasculature (S9J Fig) and inhibited vascular network formation (branching points) and sprouting (red-broken circles) in the OPL (S9K Fig). These data suggest that VEGFR3 is activated in the receptor kinase activity-independent manner during deep plexus development.

Because ECM-mediated signals upregulated the Snail-VEGFR3 axis, we expected that Snail-mediated VEGFR3 upregulation may facilitate angiogenic sprouting and migration toward the fibrin gel. Results from the fibrin sprouting assay suggest that Snail overexpression promotes angiogenic sprouting with regard to sprout number, length, and branch points. Knockdown of VEGFR3 reduced Snail-mediated sprouting (Fig 7E).

Overall, our data suggest that Snail-mediated VEGFR3 expression plays a crucial role in sprouting angiogenesis, particularly in the process of deep capillary plexus formation

## Discussion

**Fig 8:**
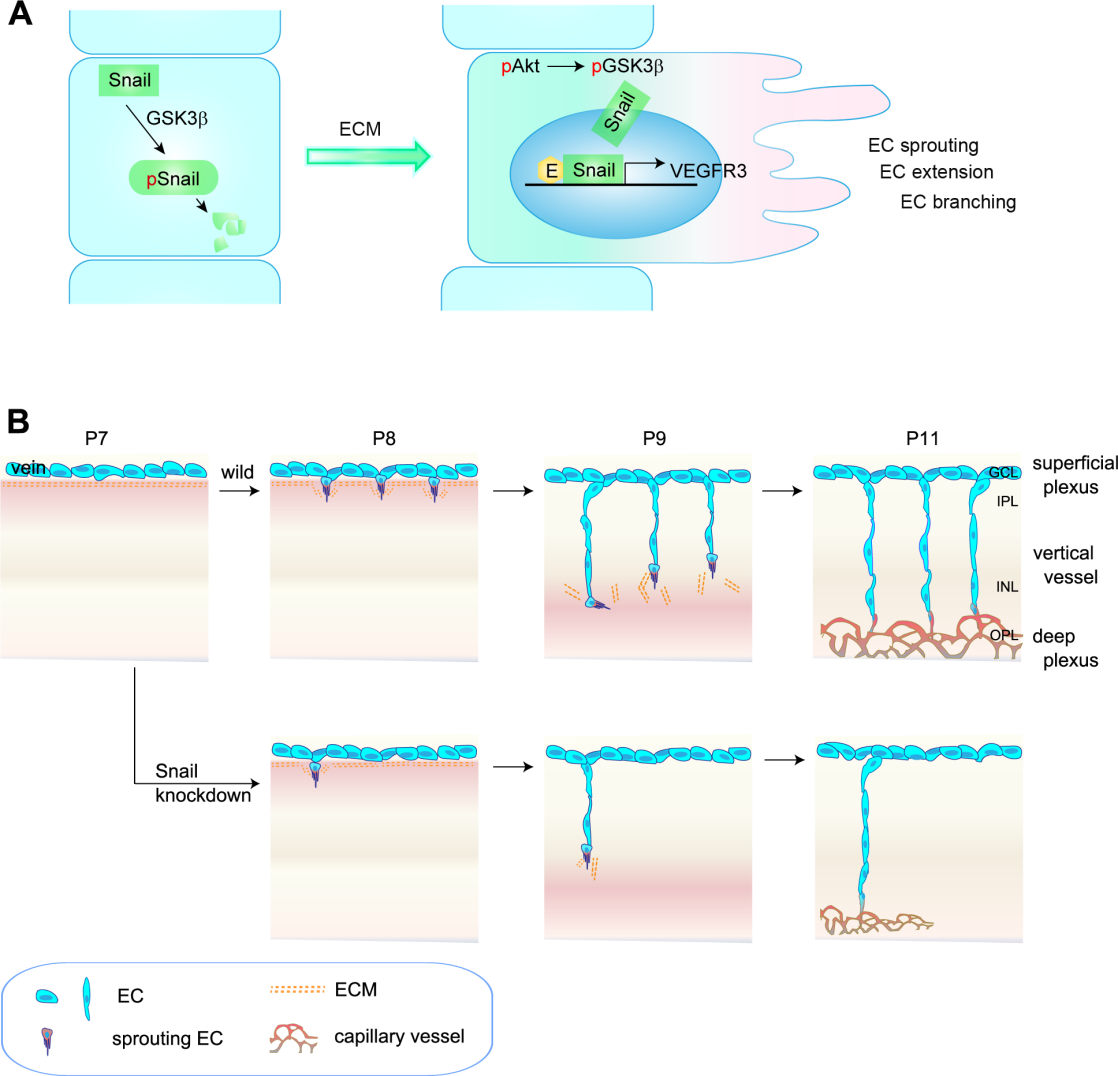
Proposed model of capillary branching morphogenesis in postnatal mice. (A) Outline of Snail stabilization by ECM-mediated signaling. Snail is rapidly degraded by the GSK3β-dependent proteosomal system. On exposure of ECs to ECM, they activate Akt, which can suppress GSK3β-dependent system by phosphorylating GSK3β (pGSK3β). This process stabilizes Snail by releasing it from GSK3β system. Thereby, the formation of Snail-Egr-1 complex promotes VEGFR3 expression by binding to the *VEGFR3* promoter region to facilitate EC morphogenesis, such as EC sprouting, extension, and branching. pSnail, phosphorylated Snail by GSK3β; pAkt, Akt phosphorylation; E, Egr-1; EC, endothelial cell. (B) Capillary branching morphogenesis is controlled by Snail. In P7–P8 mice, venous ECs in the superficial plexus start to extend capillary branching toward the deep retina in response to tissue needs. The sprouting ECs at the border between the GCL and IPL are exposed to ECM, which subsequently contributes to Snail induction and stabilization, followed by enhanced VEGFR3 expression. Snail/VEGFR3-expressing ECs vertically migrate toward deep retina. At P9–P11 mice, vertically migrating ECs reach in the boundary of INL and turn sideways to form the deep capillary plexus in the OPL region. Snail knockdown attenuates the initiation of EC sprouting, which subsequently impairs the formation of the deep capillary plexus.

Findings from this study show that (a) Snail was induced in angiogenically activated ECs in the postnatal retinal vasculature via ECM signaling; (b) the Snail-Egr-1 complex upregulated *VEGFR3* mRNA; and (c) Snail knockdown attenuated the formation of the deep vascular plexus by impairing vertical sprouting and affecting VEGFR3 expression. Collectively, the data demonstrate that a Snail-VEGFR3 axis contributed to the extension of capillary vessels and venous vessels (Fig 8).

The regulated expression of genes to sense and interpret intrinsic and extrinsic changes is needed for functional and efficient sprouting of ECs in space and time [32,33]. Genetic experiments have established VEGFR3 as a negative regulator that interferes with VEGFR2 and NRP complex formation in the superficial plexus, which highly expresses VEGFR2 [7]. Interestingly, VEGFR3 has also been suggested to positively regulate angiogenesis under certain conditions, including ECM signaling and VEGFR2-independent conditions [5,7,31]. In addition, VEGFR3 is strongly expressed in capillaries of interductal breast tumors and in small vessels that are incompletely covered by perivascular cells, thus demonstrating a role of proangiogenic VEGFR3 in capillary extension [34]. Studies in zebrafish have demonstrated VEGFR3-dependent hyperbranching in the absence of Dll4 and suggested that VEGFR3 is indispensable for venous angiogenesis [8]. Thus, the differential expression patterns of VEGFR2, VEGFR3, and Dll4 may be related to variations in vessel patterning. As a result, we sought to investigate whether the differential expression pattern and functionality of VEGFRs may be due to changes in the microenvironments in which vascular branching morphogenesis occurs. In developing retina vasculature, the vertical vessels extend and migrate from the GCL (rich in nuclei) to IPL (rich in neurites), and they seem likely to experience microenvironments that may differ with regard to ECM components and growth factors [3,21]. Our findings show that ECM components played an important role in VEGFR3 expression. Moreover, VEGFR3 expression was high in vertical vessels and the deep capillary plexus. We and others have shown that VEGFR2 is strongly expressed in neurons, but not ECs, in the deep retina [35]. Moreover, Okada *et al*. have shown that the neuronal expression of VEGFR2 can inhibit vertical angiogenesis by titrating soluble VEGFA [35]. Based on these reports, we assumed that VEGFR3 expression in deep retinal ECs could contribute to angiogenesis, whereas VEGFR2 expression in neurons could limit retinal angiogenesis. Our data show that stable siSnail mice exhibited reduced VEGFR3 expression and defective vertical vessels, demonstrating that the Snail-VEGFR3 promoted angiogenesis under conditions involving ECM exposure and low levels of VEGFR2.

The formation of the deep plexus occurs in mice at P8 when venous vessels in the superficial plexus sprout vertically and extend (Fig 8B) [2,3]. The vertical sprouting begins in the center of the retina (optic stalk) and expands toward the margin. The extension of vertical vessels from the superficial plexus penetrates the retina to reach the boundary of the OPL. In the OPL, the vessels sprout and interact sideways to form the capillary network. Several reports have suggested a potential involvement of perivascular or extravascular cells in the formation of the deep retina plexus. Neuroglial expression of VEGFA in the INL seems to regulate the timing for vertical sprouting [21,35]. The border of the INL seems to express VEGFA for induction of the deep plexus. Macrophages have also been shown to regulate deep vessel branches [7,36,37]. M2-type macrophages can promote angiogenesis by secreting growth factors, such as VEGFC, whereas M1-type macrophages can inhibit angiogenesis by initiating programmed EC death and engulfing dying cells [38]. In particular, the macrophage can act as a bridge between two tip cells in the sprouts to establish a vascular network via VEGFR3 in the process of sprout anastomosis [7]. However, it is suggested that macrophages can also have anti-angiogenic effects in the deep vasculature. When vertical sprouts are close to initiating the deep plexus in the INL and OPL, macrophages are in close proximity to the vertical sprouts and can inhibit their branching. Wnt and VEGFR1 pathways are involved in the inhibition of vertical branching in the developing retina [36]. In addition, the production of the Notch ligand, Delta-like 1, by extravascular cells is essential for endothelial sprouting toward the deep retina [39]. Further insights into the development of the deep plexus can be gained by using adhesion molecules and ECM components. R-cadherin levels are increased in the border of the INL region, where the deep plexus is formed [40]. The ECM component, fibronectin, may affect the vascular phenotype, as it accumulates around sprouting vessels [41]. We also show through *in vitro* and *in vivo* studies that ECM-induced Snail plays an important role in the initiation process of venous sprouting from the superficial plexus in the deep retina vasculature. Regarding the downstream of ECM, it is reported that ECM-activated Src and Rho initiate capillary morphogenesis via Snail *in vitro* [42]. In addition, *in vitro* study shows that integrin-mediated VEGFR3 is activated in a c-Src-dependent manner, and this activity of VEGFR3 is distinct from that of ligand-induced receptor kinase activity that is inhibited by MAZ51 [9]. We also found in this study that MAZ51 did not attenuate, but the Src family inhibitor, PP2, delayed and reduced the deep plexus. Although further studies are required to elucidate the role of endothelial c-Src in the deep plexus, our results suggest that the Snail-mediated increase in VEGFR3 can facilitate and augment sprouting vessels by c-Src. Hence, the orchestrated combination of the ECM-mediated induction of the endothelial Snail-VEGFR3 axis, Wnt-VEGFR1 pathway, and neuronal VEGFR2 may serve as triggering cues for the formation of the deep capillary plexus.

The vascular capillary is flexible and dynamic. It repeatedly appears or disappears in response to different physiological and pathological states. For example, both neuronal synaptic activity and ischemic diseases in the retina and brain are deeply correlated with the dynamics of vascular capillaries. Given that Snail and Egr-1 are increased in ischemic conditions [28,43], the rapid induction of Snail and Egr-1 in veins and venules in response to local changes of the retina and brain can trigger the initiation of vessel sprouting. This may be followed by VEGFR3 upregulation, which induces EC sprouting and capillary morphogenesis. Furthermore, the induction and extension of tumor capillary vessels may be mediated via Snail and VEGFR3 in ductal breast tumors, because normal resting vessels do not express Snail and VEGFR3 [19,34]. Therefore, the spatiotemporal expression pattern of the Snail-VEGFR3 axis in response to local needs is likely to play a crucial role in transient capillary formation.

Many studies have focused on the contribution of the Snail family to epithelial morphogenesis under physiological conditions and to the EMT phenomenon under pathological conditions [12,44]. Despite similarities between epithelial cells and ECs in their morphogenetic processes, the role of the Snail family in ECs has barely been studied. In our study, Snail-deficient ECs were unable to sprout and migrate. Similar results have been recently reported [18]. Epithelial branching morphogenesis requires Snail for branching initiation during mammary epithelial branching [13]. Furthermore, epithelial branching initiation is thought to be triggered by mesenchymal markers, such as Snail and vimentin, at branch sites. This is a potential role of partial EMT in branching morphogenesis. However, vascular branching may not be related to this process. The EC itself has been shown to exhibit mesenchymal-like characteristics and express high levels of vimentin [45]. Moreover, ECs can easily shuffle between the tip and stalk cells in angiogenic leading vessels. However, the possible presence of endothelial-mesenchymal transition (EndMT) in angiogenic processes has also been suggested [46]. Mice with endothelial-specific disruption of cerebral cavernous malformation-1 undergo EndMT by upregulating transforming growth factor-β and BMP signaling. Hence, whether our findings regarding Snail expression at endothelial branch points are akin to EndMT will require further analysis.

Overall, we propose that Snail contributes to capillary formation through the initiation of venous sprouting (Fig 8). The induction and stabilization of Snail in response to local changes could promote morphological changes via the loss of EC junctions (e.g. ZO-1 and Occludin), loss of EC polarity, and degradation of the basement membrane (e.g., MT-MMPs) [18]. Snail also increased VEGFR3 expression to initiate EC sprouting, which subsequently led to the formation of the deep vascular network. Our findings provide mechanistic insights into the induction of EC branching events by factors that are specifically and transiently regulated within the microenvironment to enforce vascular branching morphogenesis, such as capillary morphogenesis.

## Materials and Methods

### Cell culture

HUVECs were isolated from human umbilical cord veins by collagenase treatment. Cells at passages 2–7 were used and cultured in EC growth medium (EGM)-2 supplemented with 10% fetal bovine serum (FBS). HRECs were purchased from Applied Cell Biology Research Institute (Kirkland), and passages 2–7 were used for experiments. HRECs were grown in EC basal medium (EBM-2) containing the EGM-2 kit (Clonetics, Lonza Walkersville) and 10% FBS. Cells were transiently transfected using lipofectamine or the lipofectamine LTX_Plus system (Invitrogen). For ECM experiments, ECs were reseeded at a density of 2–2.5×10^4^ cells/cm^2^ in poly-L-lysine (20 μg/mL), (fibronectin (20 μg/mL)- or collagen type I (20 μg/mL)-coated dishes in EBM-2 supplemented with 1–2% FBS for the indicated time points. Small-interfering Egr-1 (siEgr-1; 5’-GUGCAAUUGUGAGGGACAU-3’) was from Bioneer Corporation (Korea). Other siRNAs were ON-TARGET plus SMART pool siRNAs that were designed by Dharmacon, Inc. Flag-Snail and Flag-Snail (6SA) were gifts from Mien-Chie Hung (Addgene plasmids #16218 and #16221, respectively). Flag-Snail (6SA) has six mutation sites (S974A, S101A, S108, S112A, S116A, and S120A). Recombinant human VEGFA 165 was from Koma Biotech., LTD. Fibronectin, collagen type I, and poly-L-lysine were from Sigma, BD Biosciences, and Sigma, respectively.

### Affymetrix oligonucleotide microarray

Growth factor-reduced Matrigel (BD Biosciences) was placed on 60-mm culture dishes and polymerized for 0.5 h. HUVECs (1×10^6^ cells) were plated on the layer of Matrigel and cultured. Total RNA was isolated and hybridized to the HG-U133A 2.0 microarray (Affymetrix), according to the manufacturer’s protocol. Full data sets are available online (GRE accession number: GSE 12891).

### Quantitative RT-PCR

Total RNA was purified using a TRIzol reagent kit (Invitrogen). Semi-quantitative RT-PCR was performed with 2x Maxima SYBR, as described in the manufacturer’s manual (Thermo Scientific). The primers used for amplification were as follows: *Snail*, 5’-CCTCAAGATGCACATCCGAAGCCA-3’ and 5’-AGGAGAAGGGCTTCTCGCCAGTGT-3’; *Slug*, 5’-CCCCCATGCCATTGAAGCTGA-3’ and 5’-GCGCCCAGGCTCACATATTCC-3’; *Egr-1*, 5’-TGACCGCAGAGTCTTTTCCT-3’ and 5’-TGGGTTGGTCATGCTCACTA-3’; *VEGFR1*, 5’-TCCTTTGGATGAGCAGTGTG-3’ and 5’-AGCCCCTCTTCCAAGTGATT-3’; *VEGFR2*, 5’-CCAGTCAGAGACCCACGTTT-3’ and 5’-TCCAGAATCCTCTTCCATGC-3’; *VEGFR3*, 5’-TTCCTGGCTTCCCGAAAGT-3’ and 5’-AGGCCAAAGTCACAGATCTTCAC-3’; glyceraldehyde 3-phosphate dehydrogenase (*GAPDH*), 5’-ATGGGGAAGGTGAAGGTCG-3’ and 5’-GGGGTCATTGATGGCAACAATA-3’.

### Western blot

ECs were washed with cold phosphate-buffered saline (PBS) and harvested in radioimmunoprecipitation assay (RIPA) buffer supplemented with 50 mM β-glycerolphosphate, 0.1 mM sodium orthovanadate, 1 mM dithiothreitol, and a protease inhibitor cocktail. The amount of protein in each sample was measured using the bicinchoninic acid assay kit (Thermo Scientific), and the proteins were separated by sodium dodecyl sulfate-polyacrylamide gel electrophoresis (SDS-PAGE). Immunoblotting was performed with antibodies to Snail (Cell Signaling Technology, Inc.), Slug (Cell Signaling Technology, Inc.), VEGFR3 (Santa Cruz Biotechnology), VEGFR2 (Cell Signaling Technology, Inc.), phosphorylated Erk1/2 (p-Erk1/2; Cell Signaling Technology, Inc.), phosphorylated Akt (p-Akt; Cell Signaling Technology, Inc.), Egr-1 (Santa Cruz Biotechnology), and β-Actin (Santa Cruz Biotechnology).

### Immunoprecipitation

ECs were seeded at a density of 2–2.5×10^4^ cells/cm^2^ on fibronectin-coated dishes. After 2 h, cells were harvested with a buffer containing 0.5% NP-40, 50 mM Tris-Cl (pH 8.0), 150 mM NaCl, 10% glycerol, 1.5 mM MgCl_2_, 50 mM β-glyceraldehyde, 50 mM NaF, 0.1 mM Na_3_VO_4_, 1 mM DTT, and a protein inhibitor cocktail. If necessary, 0.5 mM ZnCl_2_ and/or 1 mM EDTA was added. The lysates were immunoprecipitated with IgG or anti-Egr-1 antibody (Santa Cruz Technology) at 4°C overnight. On the next day, protein A/G slurry beads (30 μl) were added for 2 h. The beads were spun and re-suspended with sample buffers and run on SDS-PAGE along with the 5% input sample and transferred to polyvinylidene fluoride membranes. Proteins were detected with the anti-Snail (Millipore clone 10H4.1) or anti-Egr-1 antibody. For VEGFA experiments, ECs were exposed to 30 ng/mL VEGFA for the indicated time points. EC lysates were immunoprecipitated with anti-Snail antibody (Cell Signaling Technology, Inc.) and then probed with the anti-Egr-1 antibody (Santa Cruz Biotechnology).

### Luciferase assay

ECs were plated on a gelatin-coated dish and transfected with 500 ng *VEGFR3* promoter-reporter constructs and 50 ng pRL-TK using Lipofectamine LTX-plus reagents (Invitrogen). In some experiments, ECs were transfected with siRNAs and then re-transfected with the *VEGFR3* promoter-reporter and pRL-TK plasmid after 4 h. On the next day, ECs were cultured in fresh EBM-2 containing 1% FBS for 4 h, reseeded at a density of 2–2.5×10^4^ cells/cm^2^, and cultured on ECM-coated dishes for another 8–16 h. The cells were lysed using passive lysis buffer (Promega), and luciferase activity was determined using a dual luciferase assay system (Promega).

### Site-directed mutagenesis

A human *VEGFR3* reporter (HPRM21111-PG02) containing the 1.3-Kb promoter region of *VEGFR3* was purchased from GeneCorpoeia, Inc. The *VEGFR3* promoter was subcloned into the pGL3-basic luciferase reporter plasmid (Promega). *VEGFR3* reporters containing mutations in the region of the putative E-box region was generated using the QuickChange II Site-Directed Mutagenesis kit (Agilent Technology), according to the manufacturer’s instructions.

### ChIP assay

The ChIP assay was performed using the ChIP kit (Millipore), according to the manufacturer’s instructions. SiSnail-transfected HUVECs were exposed to formaldehyde (1% final concentration) to cross-link their genomic DNA and protein. The cells were harvested, lysed, and sonicated to generate 0.3–1.0-kb DNA fragments. After centrifugation, the cleared supernatant was incubated with the anti-Snail antibody (Abcam, ab85931) or Immunoglobulin G for immunoprecipitation. The primers used for amplification were as follows: 5’-GGAAAGAAAGGACGGAAAAGAGC-3’ and 5’-GCTGCGCGTGGGTCCGA-3’; 5’-GCTCCCCTTTGCCCACCAG-3’ and 5’-CCACAGTCGCAGGCACAGC-3’. PCR amplification was carried out under the condition of 95°C (60 sec), 60°C (30 sec), and 72°C (30 sec) for 40–45 cycles. Amplified DNA was separated on a 1.5% agarose gel and visualized with ethidium bromide.

### Vascular network formation assay

HUVECs were loaded onto Matrigel on a 24-well culture dish at a density of 1.5×10^5^ cells/well in EGM-2 supplemented with 10% FBS. Cells were photographed at the indicated time points. Cells were also transfected with siCon (40–80 μM) or siSnail (40–80 μM). For quantitative RT-PCR or western blot analysis, cells were recovered from Matrigel using the Cell Recovery solution (BD Bioscience), and RNA or protein was isolated.

### Fibrin gel bead assay

Following transfections, HUVECs were mixed with Cytodex microcarrier beads at a ratio of 1×10^6^ cells:2500 beads. Coating was performed for 4 h in fluorescence-activated cell sorting tubes, which were shaken by pipetting every 20 min. After 24 h, the coated beads were dissolved in a solution of 2 mg/mL fibrinogen and 0.15 units/mL aprotinin in EGM-2. The solution was added to 0.625 units/mL thrombin in each well of a 24-well plate. After forming clots, 2×10^4^ WI-38 fibroblasts or fibroblast-conditioned media were loaded into each well. The medium was replaced every 2 days, and sprouting was analyzed after 7–10 days. The mean number of sprouts per bead was determined by counting the number of sprouts that originated from the cells that lined the surface of the bead, and the mean number of branch points per bead was determined by counting the number of sprout bifurcations per bead.

For single EC sprouting assays, ECs were transfected with siSnail, flag-Snail or flag-Snail (6SA). The cells were resuspended in a solution of 2 mg/mL fibrinogen, 0.15 units/mL aprotinin, and 0.625 units/mL thrombin, and then rapidly loaded on top of a precoated fibrin layer. When the fibrin gel formed clots, a solution containing a 1:1 mixture of fresh EGM-2 and WI-38 fibroblasts-conditioned medium was loaded into each well and replaced every 2 days. After 7–10 days, cells were stained with 4 μg/mL calcein AM and imaged by fluorescence microscopy. The cumulative sprout length was quantified in a minimum of 6 fields (720 μm × 530 μm), and the total length was normalized to 1000 μm.

### Whole flat-mount and immunochemical assays in the postnatal retina

The eyeballs of C57/BL6 mice were enucleated and fixed in 4% paraformaldehyde (PFA) for 1 h or 1% PFA for 30 min to stain retinal vessels or corneas, respectively. In whole flat-mount assays for Snail staining, retinas were dissected, post-fixed in methanol, and permeabilized with 0.5% saponin and 0.25% BSA in PBS overnight. The retinas were incubated with anti-Snail antibody (Millipore, Clone 10H4.1) overnight. After washing with 0.25% saponin in PBS, retinas were incubated with Alexa Fluor 488-goat anti-mouse IgM and 594-conjugated Isolectin GS-iB4 solution at 4°C overnight. For VEGFR3 or CD29 stainings, a detergent was minimally used in the process of blocking, washing, and incubation with anti-VEGFR3 (Abcam, ab51874; R&D, AF743) antibodies. The retinas were flat-mounted on slides using fluorescent mounting medium. Images were captured with Carl Zeiss confocal microscopes (LSM 510 META or LSM 700). For immunochemical assays, mouse eyeballs were fixed in 4% PFA overnight, incubated in 15% sucrose, and transferred to 30% sucrose at 4°C until they sank. The eyeballs were transferred to Optimal Cutting Temperature compound-embedding medium, sectioned (8–12 μm), and then stored at -70°C.

### Animal studies

All mice were maintained in a laminar air flow cabinet under specific pathogen-free conditions. All facilities were approved by the Association of Assessment and Accreditation of Laboratory Animal Care, and all animal experiments were conducted under institutional guidelines that were established for the Animal Core Facility at Yonsei University College of Medicine (Korea, Seoul).

### *In vivo* Matrigel plug assay

The pGFP-C-shLenti mouse Snail clone (A–D) sets were purchased from Origene Technologies, Inc. Of these, pGFP-C-shLenti mouse Snail clones A and B (referred as to shSnail #1 and #2 in this study) were selected. The lentiviruses were collected and concentrated after checking for GFP-positive staining in 293T cells. C57/BL6 mice (7 weeks old) were injected subcutaneously with 0.6 mL Matrigel containing GFP-shLenti Control or GFP-shLenti Snail. After 6 days, the skin of each mouse was pulled back to expose the Matrigel plug, which remained intact. To identify infiltrating mouse ECs, immunohistochemistry was performed with the anti-CD31 antibody (BD Biosciences).

### *In vivo* retina study

For *in vivo* siRNA injections, mouse siSnail sequences were selected among four sets of mouse siSnail (Dharmacon; ON-TARGETplus Mouse SnailLU-062765) and manufactured as *in vivo* siSTABLE mSnail (siSnail) by Dharmacon (5’-CAAACCCACUCGGAUGUGAUU-3’). C57/BL6 mice were injected intraperitoneally with 4 mg/kg siSnail or scrambled siRNA (siCon) from P6–P7 or from P7–P10. Mice were sacrificed at P8 or P11, and enucleated eyes were processed for whole flat-mount staining. Each experiment was performed with three pups per group and repeated four times. For shLenti Snail virus injections, shCon or shSnail#2 was intraperitoneally injected at P6 and P7, and the mice were sacrificed at P8. Enucleated eyes were processed for whole flat-mount staining. Each experiment was performed with three or four littermates per group and repeated three times. For the pharmacological inhibition of VEGFR3 kinase activity and c-Src activity *in vivo*, 10 mg/kg MAZ51 (Sigma) or 10 mg/kg PP2 (Sigma) were intraperitoneally injected. To analyze the superficial plexus, MAZ51 was injected at P4 and P5, and mice were sacrificed at P6. To analyze the deep plexus, MAZ51 or PP2 was injected from P7 to P9 and P10, and then mice were sacrificed at P10 or P11. The retinal vessel area, radial length, branching points, and vertical vessels were measured. All pups were weighed before experiments. Littermates with identical weights for each experiment were used. Each experiment was repeated three times.

### Statistical analysis

The vessel length and area were determined by using Multi Gauge Fuji film (Tokyo, Japan). Data were presented as mean ± standard deviation or mean ± standard error. Statistical comparisons between groups were performed using one-way analysis of variance, followed by the Student’s *T* test. All experiments were performed at least three times, and representative results were shown.

## Supporting Information

S1 Fig Snail is upregulated during *in vitro* vascular network formation. (**A** and **B**) DNA microarray analysis showing the expression of epithelial-mesenchymal transition (EMT)-related genes. Human umbilical vein endothelial cells (HUVECs) were loaded on Matrigel and cultured for the indicated time points. At the indicated time points, phase-contrast images were taken (**A**). RNA was extracted and applied to the Affymetrix oligonucleotide array (**B**). On the basis of the Affymetrix oligonucleotide array (GSE12891), the relative expression levels of the indicated genes were calculated at 0.5, 1, 2, 4, and 8 h against 0 h. *Snail1* and *Slug* were dramatically increased at 0.5 h and 1 h, after which their levels sharply dropped. The expression levels of other EMT-related genes (*Zeb1/2* and *Twist1/2*) were unchanged.
(TIF)

S2 Fig Snail expression in sprouting vessels. (A) Illustration of the developing retinal vessel from the superficial plexus to the deep plexus. P0, postnatal day 0. GCL, the ganglion cell layer; IPL, the inner plexiform layer; INL, the inner nuclear layer; OPL, the outer plexiform layer. (B) Confocal images showing Snail expression in the sprouting vessels in the front region of the growing vessels in mice at P5. iB4, isolectin B4. (C) Confocal images were collected in 1-μm z-stacks in the xz and yz axes at P5. Two-dimensional planes show Snail immunoreactivity (green) in leading tip cells. Z-stack images demonstrate that Snail was immunoreactive in vessel nuclei. DAPI (blue) was used to stain nuclei. (D) Confocal images of Snail immunoreactivity at P11 by whole flat-mount staining. The images were taken in the indicated regions by moving the confocal microscopic focus along the z-stack. Snail immunoreactivity (green, arrows) was found in the sprouting vessels. (E) Confocal images were collected in 1-μm z-stacks in the xz and yz axes at P11. This figure is related to **D**.
(TIF)

S3 Fig Effect of Snail on vessel sprouting in fibrin gels. **(A** and **B)** Single-suspension sprouting assays. HUVECs were embedded in fibrin gels after small-interfering RNA targeting Snail (siSnail), flag-Snail, or flag-Snail (6SA) transfection. After 7–10 days, HUVECs were labeled with calcein AM and photographed using fluorescence microscopy. Arrows indicate the termination of EC sprouts. Arrow heads indicate branching points. The cumulative sprout length, tubes, and branching points were calculated and normalized (graphs). *, p<0.01.
(TIF)

S4 Fig Snail regulates *VEGFR3* promoter activity in HUVECs. (A) Exposure of HUVECs to the ECM induces VEGFR3 expression. Quantitative reverse transcription-polymerase chain reaction (RT-PCR) analysis showing VEGFR3 expression in HUVECs that were cultured on G-, FN-, or CI-coated dishes for 16 h. G, gelatin; FN, fibronectin; CI, collagen type I. *, p<0.05. (B) *VEGFR3* promoter activity after the knockdown of Snail or early growth response protein-1 (Egr-1). HUVECs were transfected with siSnail or siEgr-1 in combination with a human *VEGFR3* promoter_luciferase (wildR3) reporter and then reseeded on FN-coated dishes at a density of 2–2.5×10^4^/cm^2^. After 16 h, the promoter activity was assessed. *, p<0.05. (C) *VEGFR3* promoter activity in Snail-overexpressing HUVECs. HUVECs were co-transfected with Snail and wildR3 reporter. *, p<0.05. (D) *VEGFR3* promoter activity by Notch signaling. HRECs were transfected with pGL3 or wildR3 reporters. Following 1 h pretreatments with 8 μM DAPT, cells were reseeded in the presence of DAPT on FN-coated dishes for 16 h. Notch siRNA (siNotch) was transfected in combination with wildR3, and transfected cells were then reseeded and cultured on CI-coated dishes for 16 h.
(TIF)

S5 Fig Verification of anti-VEGR3 antibodies. (A) Drawing of an eyeball in mice at P5. (B and C) Verification of anti-VEGFR3 antibodies. Whole flat-mount staining was applied to corneas at P5 to confirm the specificity of the anti-VEGFR3 antibody that was used in this study. VEGFR3 co-localized with iB4 (B) and lymphatic vessel endothelial receptor (LYVE) (C). Boxes are magnified in the below, respectively. In the cornea, lymphatic vessels (LYVE^+^ vessels) were strongly immunostained with the anti-VEGFR3 antibody. Blood vessels (iB4) were stained with the anti-VEGFR3 antibody. DAPI (blue) was used to stain nuclei. The region in the box is magnified in the below.
(TIF)

S6 Fig VEGFR3 expression in the deep capillary plexus. (A) Confocal serial images at P11 showing VEGFR3 expression in vertically descending vessels and deep capillary plexus. Whole flat-mount retina was stained with anti-VEGFR3 antibody (red) and iB4 (green). Confocal images were captured and displayed in every 1 μm z-axis from superficial plexus to deep plexus. DAPI (blue) was for nuclei. (B) Confocal image showing VEGFR3 in vertical vessel invading toward deep retina.
(TIF)

S7 Fig Snail knockdown attenuates the formation of vertical vessels. (A) Confocal images showing knockdown of Snail in stable siSnail-injected mice at P9. Whole flat-mount retinas were stained with Snail and iB4 at P9. Mice were consecutively injected intraperitoneally with siCon or siSnail at P6-P8, as described in Fig 6A (left). Images were taken in the superficial plexus (GCL), vertical vessels (IPL) and the deep plexus (OPL). Both Snail immunoreactivity and sprouting vessels were reduced in stable siSnail-injected mice. The white broken-circles indicate the vascular area. V, vein. (B) Confocal images of iB4 staining in the superficial plexus and vertical vessels of shCon- or shSnail-injected mice. Mice were consecutively injected intraperitoneally with shCon or shSnail at P6 and P7, as described in Fig 6A (left). The shSnail that was described in Fig 1F (shSnail #2) was used here. Mice were sacrificed at P8, and whole-mount retinas were stained with iB4. Images were taken in the superficial plexus and vertical vessels (left). Broken circles indicate sprouting vessels. The numbers of sprouting vertical vessels were quantified (right).
(TIF)

S8 Fig Snail knockdown downregulates VEGFR3 expression. (A) Whole flat-mount immunostainings of iB4 and VEGFR3 in the deep capillary plexus in stable siCon- or siSnail-injected mice. This figure is related to Fig 6C. Stable siSnail-transfected mice exhibited faint expression of VEGFR3 (white) as well as a defect in the deep capillary plexus (iB4, green). (B) Whole flat-mount immunostainings of VEGFR3 were enlarged from white box in **A**.
(TIF)

S9 Fig Inhibition of VEGFR3 receptor kinase activity with MAZ51 does not affect the formation of the deep capillary plexus. (A-C) Whole flat-mount immunostaining of iB4 in the superficial plexus of retinas after consecutive, intraperitoneal injections of MAZ51 at P4 and P5. MAZ51 efficiently attenuated the superficial vasculature (A) and sprouts at the vascular front (B). Yellow dots in **B** indicate sprouts. *, p<0.01. Quantification of radial length and sprouts from the retinas shown in **A** and **B** (C). MAZ, MAZ51. (D-F) Whole flat-mount immunostaining of iB4 in the deep capillary plexus of retinas after consecutive, intraperitoneal injections of MAZ51 from P7 to P10. The yellow broken-lined circle indicates the vascular area at P11 (D). The vasculature around the optic stalk that enlarged from **D** is shown (E). The total vessel area was quantified (F). (G) Whole flat-mount immunostaining of CD29 (red) in retinal vessels at P8. CD29 immunoreactivity was observed in vessels that sprouted from venous vessels. (H) Z-stack analysis showing CD29 immunoreactivity (red) in vertical sprouting vessels from the GCL to the IPL at P8. (I) Whole flat-mount immunostaining of CD29 combined with VEGFR3 immunoreactivity in vertical sprouts from the vein at P8. (J) Low magnification of whole flat-mount immunostaining of iB4 in the deep capillary plexus after consecutive, intraperitoneal injections of PP2 from P7 to P9. The yellow broken-lined circle indicates the vascular area at P10 (left). Quantification of vessel area in the deep plexus (right). *, p<0.01. (K) High magnification of whole flat-mount immunostaining of iB4 in the superficial plexus (GCL) and deep plexus (OPL) at P10. Confocal images were taken in the superficial region and the deep plexus by moving the microscopic focus down, along with z-stacks. White-broken lines indicate the position of the vein in the superficial plexus. Red-broken circles indicate vessel-free regions (left). Yellow arrows indicate interconnected vessels by sprouting (left). Quantification of branching points in the deep plexus (right). *, p<0.01.
(TIF)
