# Supplementary figures and images for: Endothelial Snail Regulates Capillary Branching Morphogenesis via Vascular Endothelial Growth Factor Receptor 3 Expression
Source: PLoS Genet. 2015 Jul 6;11(7):e1005324. doi: 10.1371/journal.pgen.1005324 (PMC4493050; doi:10.1371/journal.pgen.1005324)

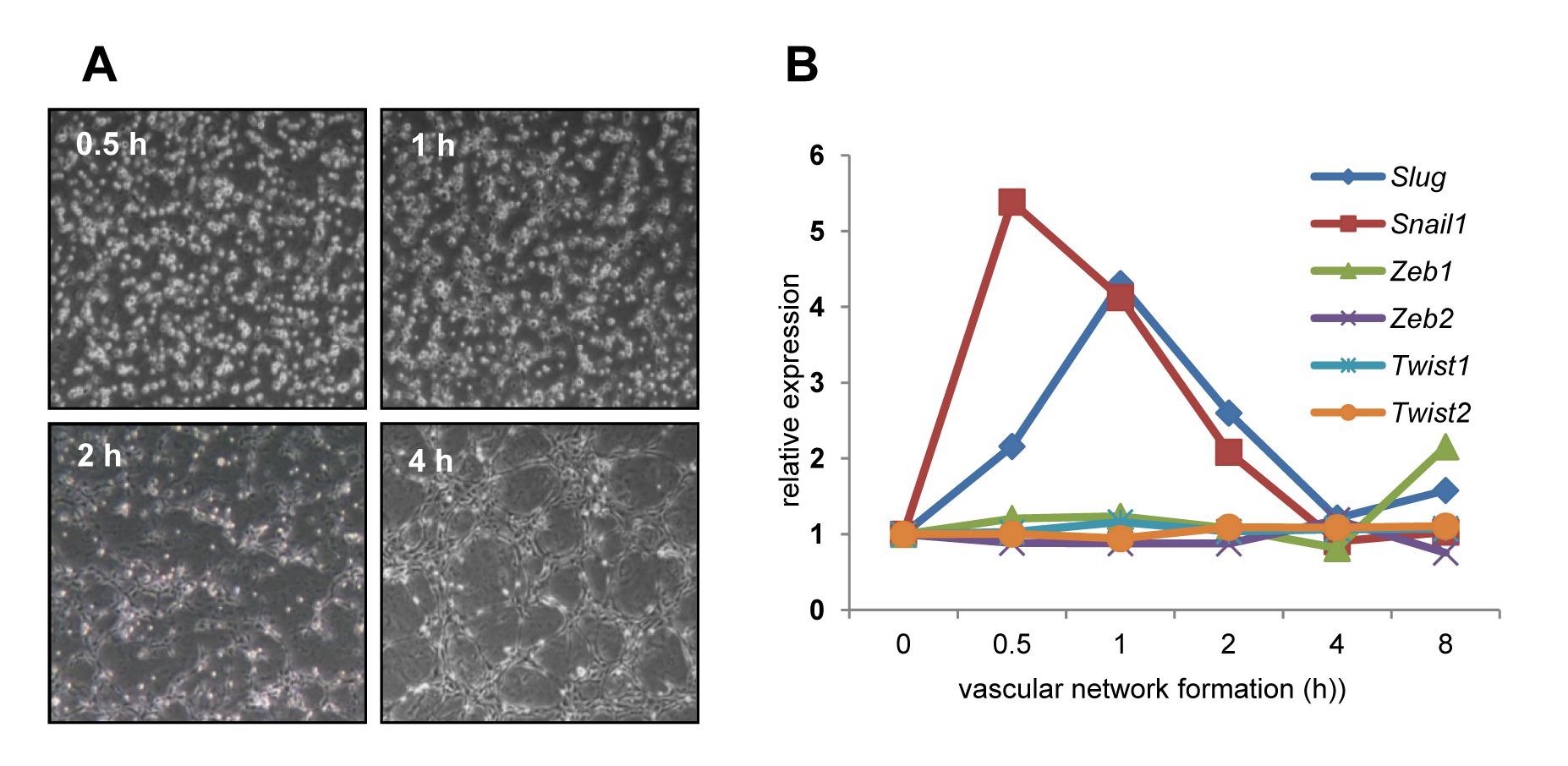

Supplement: S1 Fig — (A and B) DNA microarray analysis showing the expression of epithelial-mesenchymal transition (EMT)-related genes. Human umbilical vein endothelial cells (HUVECs) were loaded on Matrigel and cultured for the indicated time points. At the indicated time points, phase-contrast images were taken (A). RNA was extracted and applied to the Affymetrix oligonucleotide array (B). On the basis of the Affymetrix oligonucleotide array (GSE12891), the relative expression levels of the indicated genes were calculated at 0.5, 1, 2, 4, and 8 h against 0 h. Snail1 and Slug were dramatically increased at 0.5 h and 1 h, after which their levels sharply dropped. The expression levels of other EMT-related genes (Zeb1/2 and Twist1/2) were unchanged. (TIF) [file pgen.1005324.s001.tif]

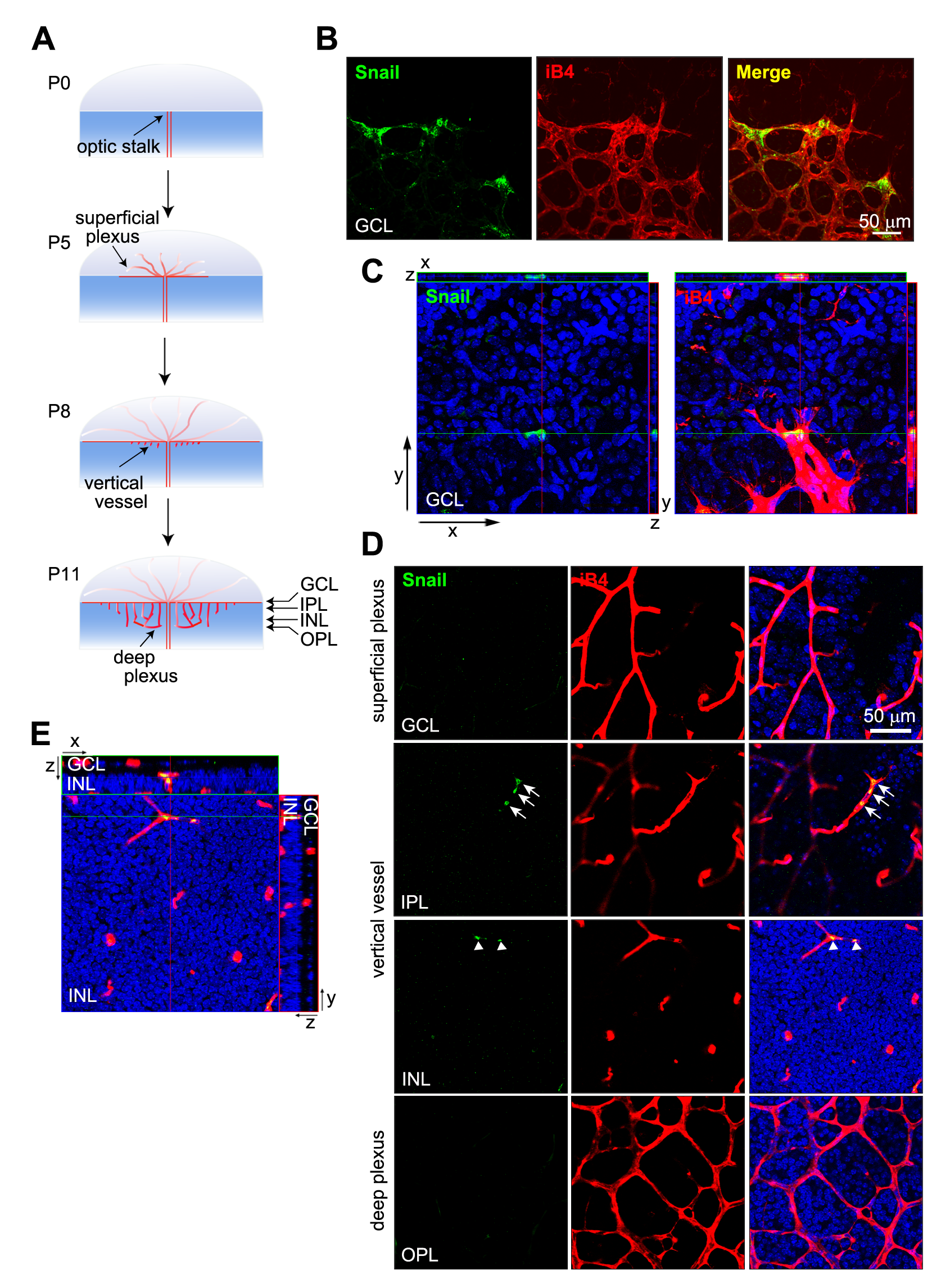

Supplement: S2 Fig — (A) Illustration of the developing retinal vessel from the superficial plexus to the deep plexus. P0, postnatal day 0. GCL, the ganglion cell layer; IPL, the inner plexiform layer; INL, the inner nuclear layer; OPL, the outer plexiform layer. (B) Confocal images showing Snail expression in the sprouting vessels in the front region of the growing vessels in mice at P5. iB4, isolectin B4. (C) Confocal images were collected in 1-μm z-stacks in the xz and yz axes at P5. Two-dimensional planes show Snail immunoreactivity (green) in leading tip cells. Z-stack images demonstrate that Snail was immunoreactive in vessel nuclei. DAPI (blue) was used to stain nuclei. (D) Confocal images of Snail immunoreactivity at P11 by whole flat-mount staining. The images were taken in the indicated regions by moving the confocal microscopic focus along the z-stack. Snail immunoreactivity (green, arrows) was found in the sprouting vessels. (E) Confocal images were collected in 1-μm z-stacks in the xz and yz axes at P11. This figure is related to D. (TIF) [file pgen.1005324.s002.tif]

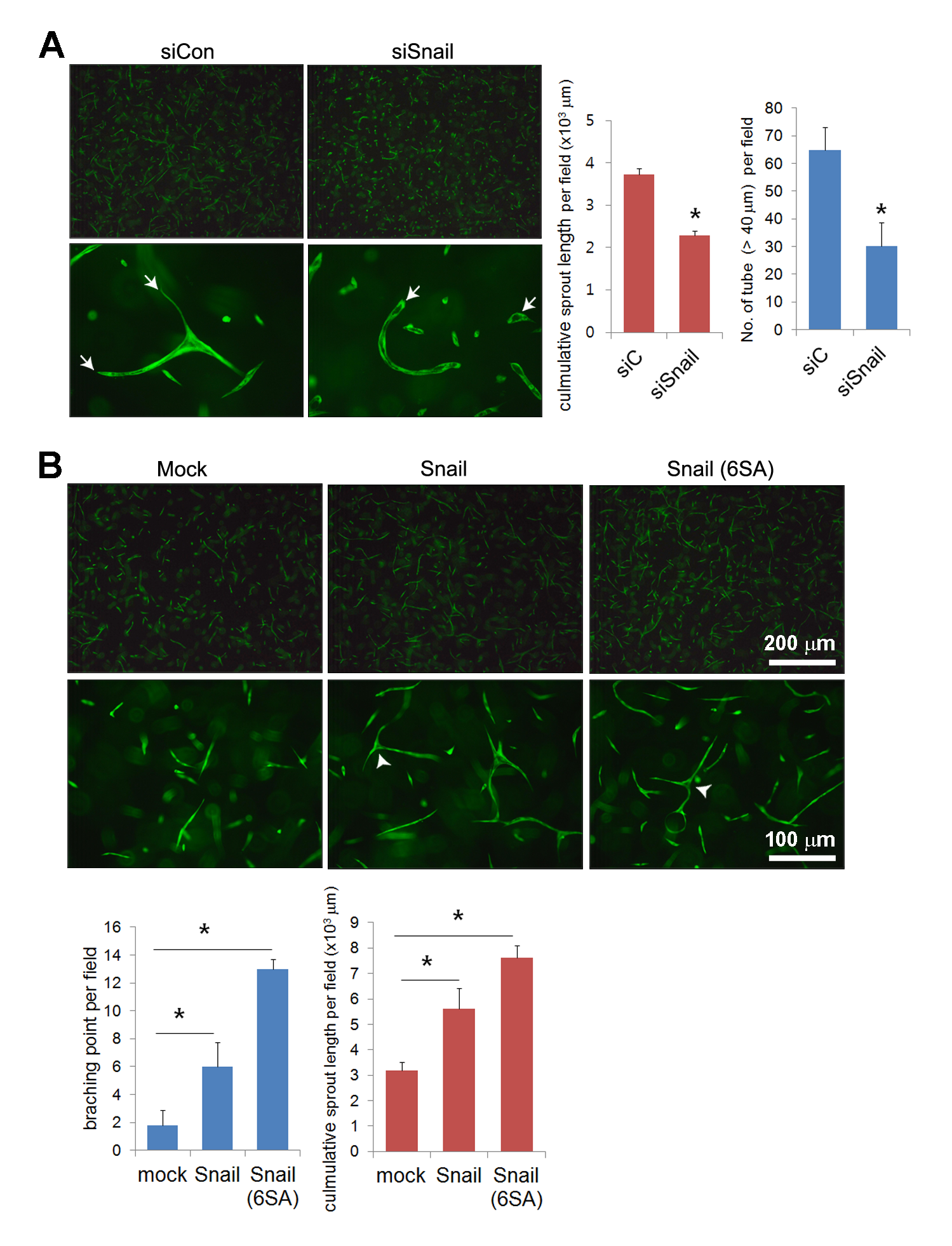

Supplement: S3 Fig — (A and B) Single-suspension sprouting assays. HUVECs were embedded in fibrin gels after small-interfering RNA targeting Snail (siSnail), flag-Snail, or flag-Snail (6SA) transfection. After 7–10 days, HUVECs were labeled with calcein AM and photographed using fluorescence microscopy. Arrows indicate the termination of EC sprouts. Arrow heads indicate branching points. The cumulative sprout length, tubes, and branching points were calculated and normalized (graphs). *, p<0.01. (TIF) [file pgen.1005324.s003.tif]

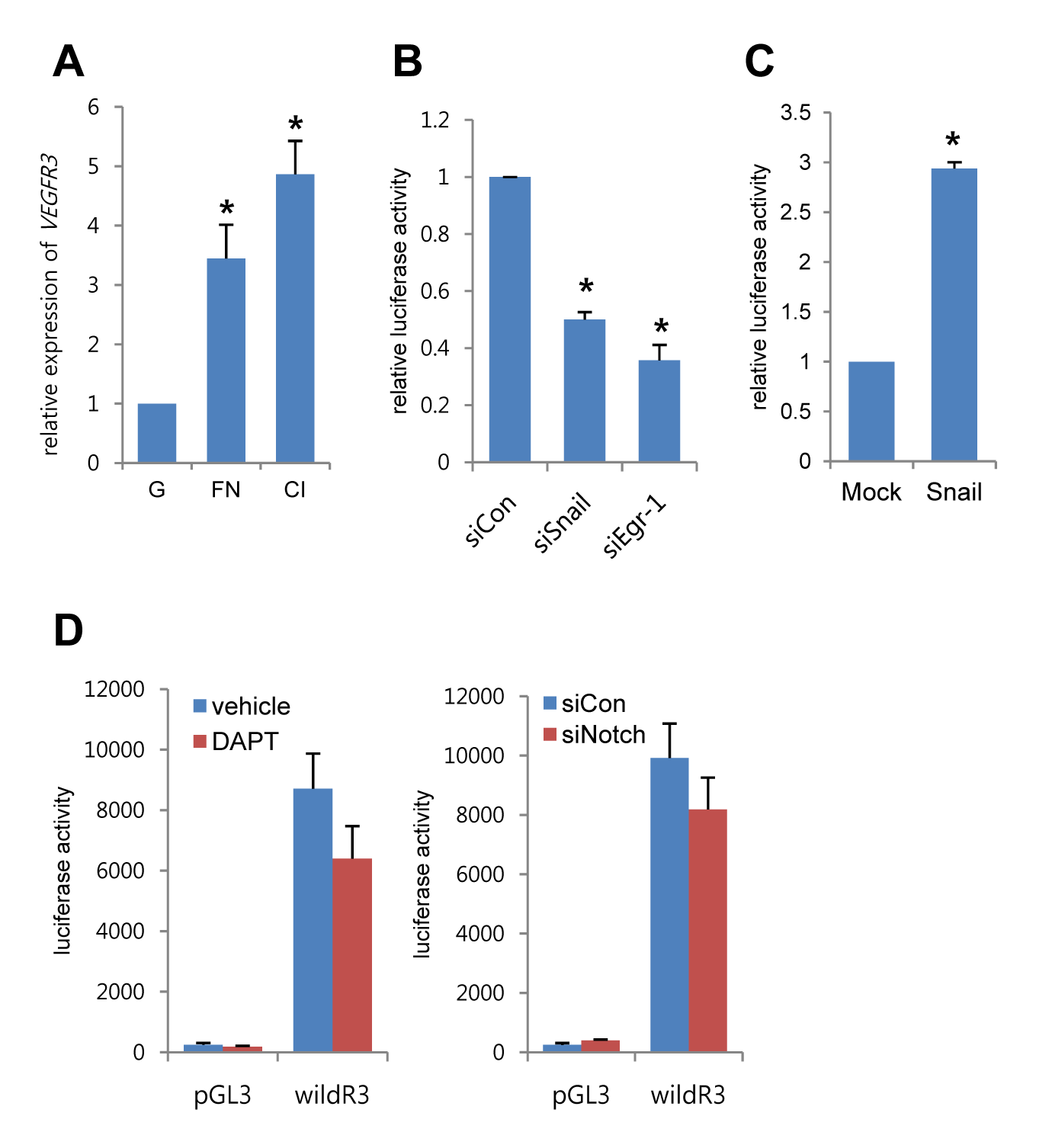

Supplement: S4 Fig — (A) Exposure of HUVECs to the ECM induces VEGFR3 expression. Quantitative reverse transcription-polymerase chain reaction (RT-PCR) analysis showing VEGFR3 expression in HUVECs that were cultured on G-, FN-, or CI-coated dishes for 16 h. G, gelatin; FN, fibronectin; CI, collagen type I. *, p<0.05. (B) VEGFR3 promoter activity after the knockdown of Snail or early growth response protein-1 (Egr-1). HUVECs were transfected with siSnail or siEgr-1 in combination with a human VEGFR3 promoter_luciferase (wildR3) reporter and then reseeded on FN-coated dishes at a density of 2–2.5×10^4^/cm2. After 16 h, the promoter activity was assessed. *, p<0.05. (C) VEGFR3 promoter activity in Snail-overexpressing HUVECs. HUVECs were co-transfected with Snail and wildR3 reporter. *, p<0.05. (D) VEGFR3 promoter activity by Notch signaling. HRECs were transfected with pGL3 or wildR3 reporters. Following 1 h pretreatments with 8 μM DAPT, cells were reseeded in the presence of DAPT on FN-coated dishes for 16 h. Notch siRNA (siNotch) was transfected in combination with wildR3, and transfected cells were then reseeded and cultured on CI-coated dishes for 16 h. (TIF) [file pgen.1005324.s004.tif]

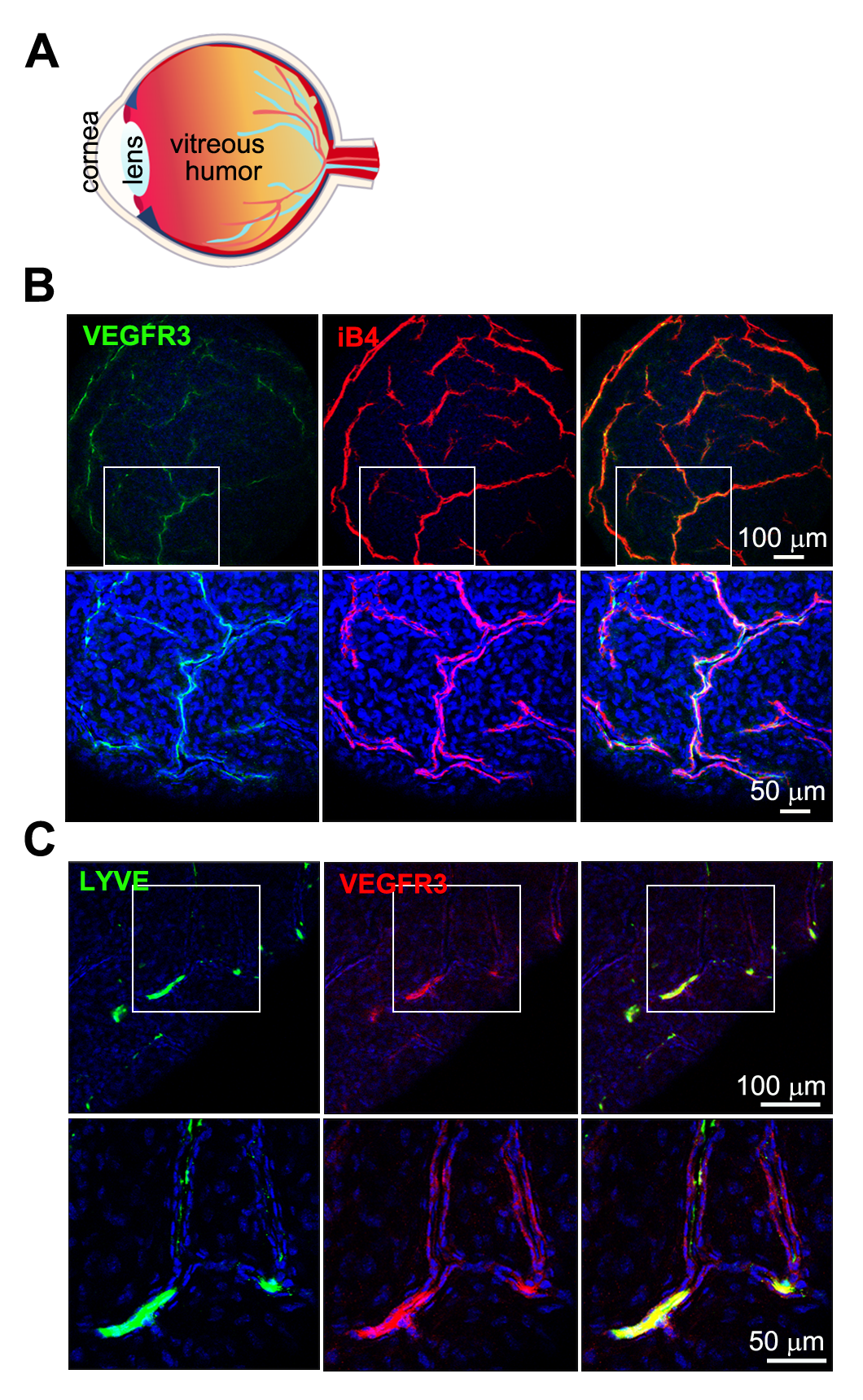

Supplement: S5 Fig — (A) Drawing of an eyeball in mice at P5. (B and C) Verification of anti-VEGFR3 antibodies. Whole flat-mount staining was applied to corneas at P5 to confirm the specificity of the anti-VEGFR3 antibody that was used in this study. VEGFR3 co-localized with iB4 (B) and lymphatic vessel endothelial receptor (LYVE) (C). Boxes are magnified in the below, respectively. In the cornea, lymphatic vessels (LYVE+ vessels) were strongly immunostained with the anti-VEGFR3 antibody. Blood vessels (iB4) were stained with the anti-VEGFR3 antibody. DAPI (blue) was used to stain nuclei. The region in the box is magnified in the below. (TIF) [file pgen.1005324.s005.tif]

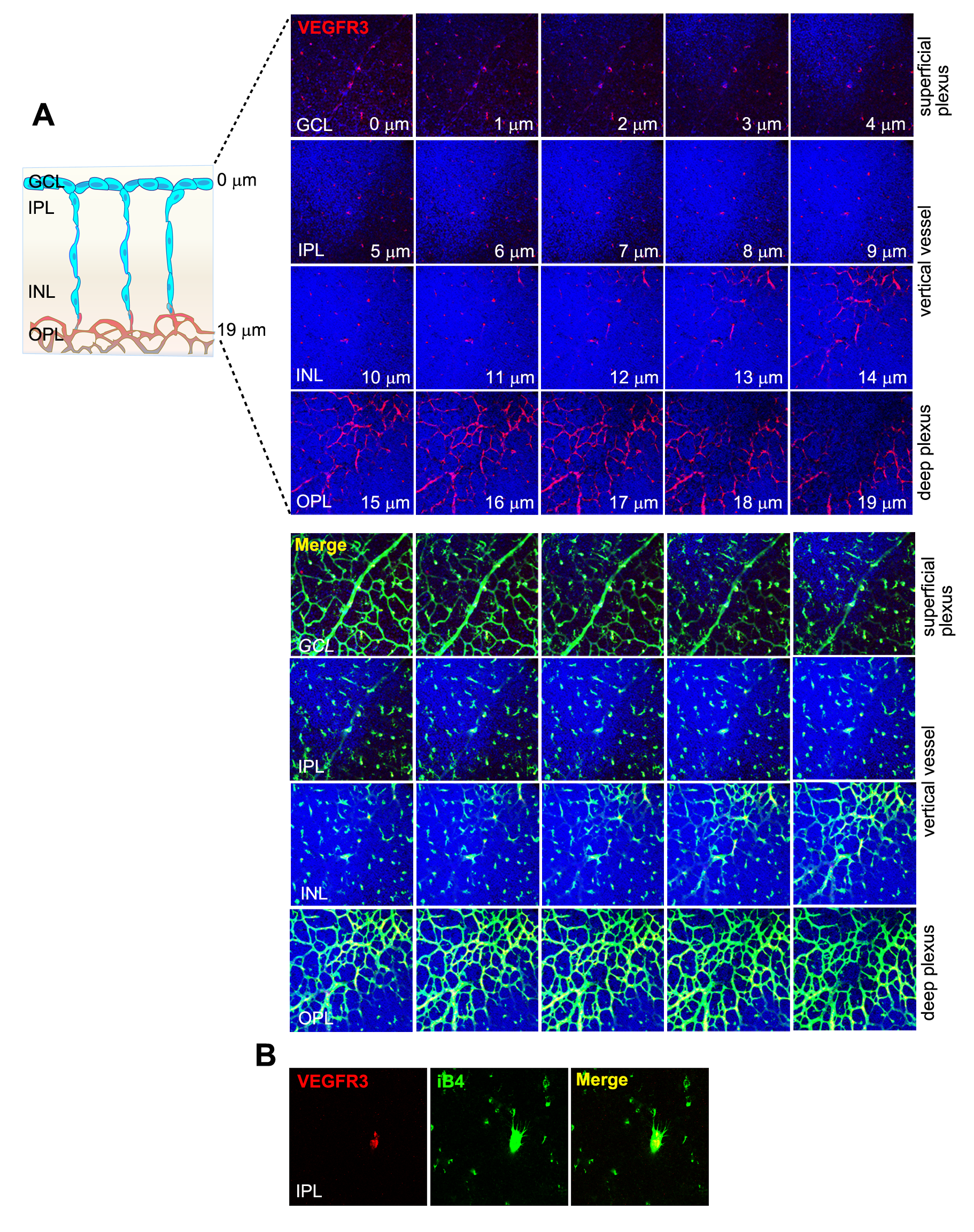

Supplement: S6 Fig — (A) Confocal serial images at P11 showing VEGFR3 expression in vertically descending vessels and deep capillary plexus. Whole flat-mount retina was stained with anti-VEGFR3 antibody (red) and iB4 (green). Confocal images were captured and displayed in every 1 μm z-axis from superficial plexus to deep plexus. DAPI (blue) was for nuclei. (B) Confocal image showing VEGFR3 in vertical vessel invading toward deep retina. (TIF) [file pgen.1005324.s006.tif]

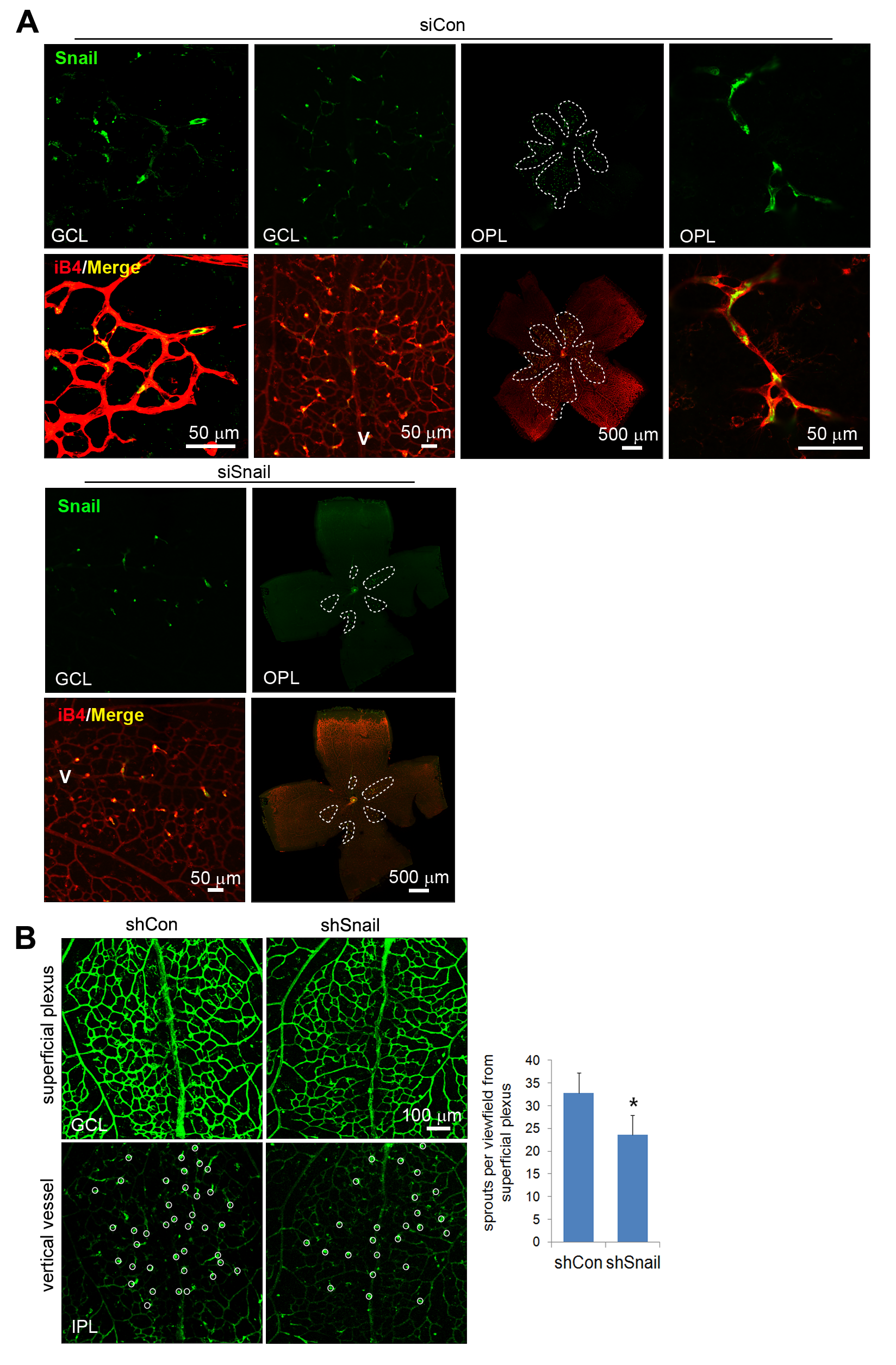

Supplement: S7 Fig — (A) Confocal images showing knockdown of Snail in stable siSnail-injected mice at P9. Whole flat-mount retinas were stained with Snail and iB4 at P9. Mice were consecutively injected intraperitoneally with siCon or siSnail at P6-P8, as described in Fig 6A (left). Images were taken in the superficial plexus (GCL), vertical vessels (IPL) and the deep plexus (OPL). Both Snail immunoreactivity and sprouting vessels were reduced in stable siSnail-injected mice. The white broken-circles indicate the vascular area. V, vein. (B) Confocal images of iB4 staining in the superficial plexus and vertical vessels of shCon- or shSnail-injected mice. Mice were consecutively injected intraperitoneally with shCon or shSnail at P6 and P7, as described in Fig 6A (left). The shSnail that was described in Fig 1F (shSnail #2) was used here. Mice were sacrificed at P8, and whole-mount retinas were stained with iB4. Images were taken in the superficial plexus and vertical vessels (left). Broken circles indicate sprouting vessels. The numbers of sprouting vertical vessels were quantified (right). (TIF) [file pgen.1005324.s007.tif]

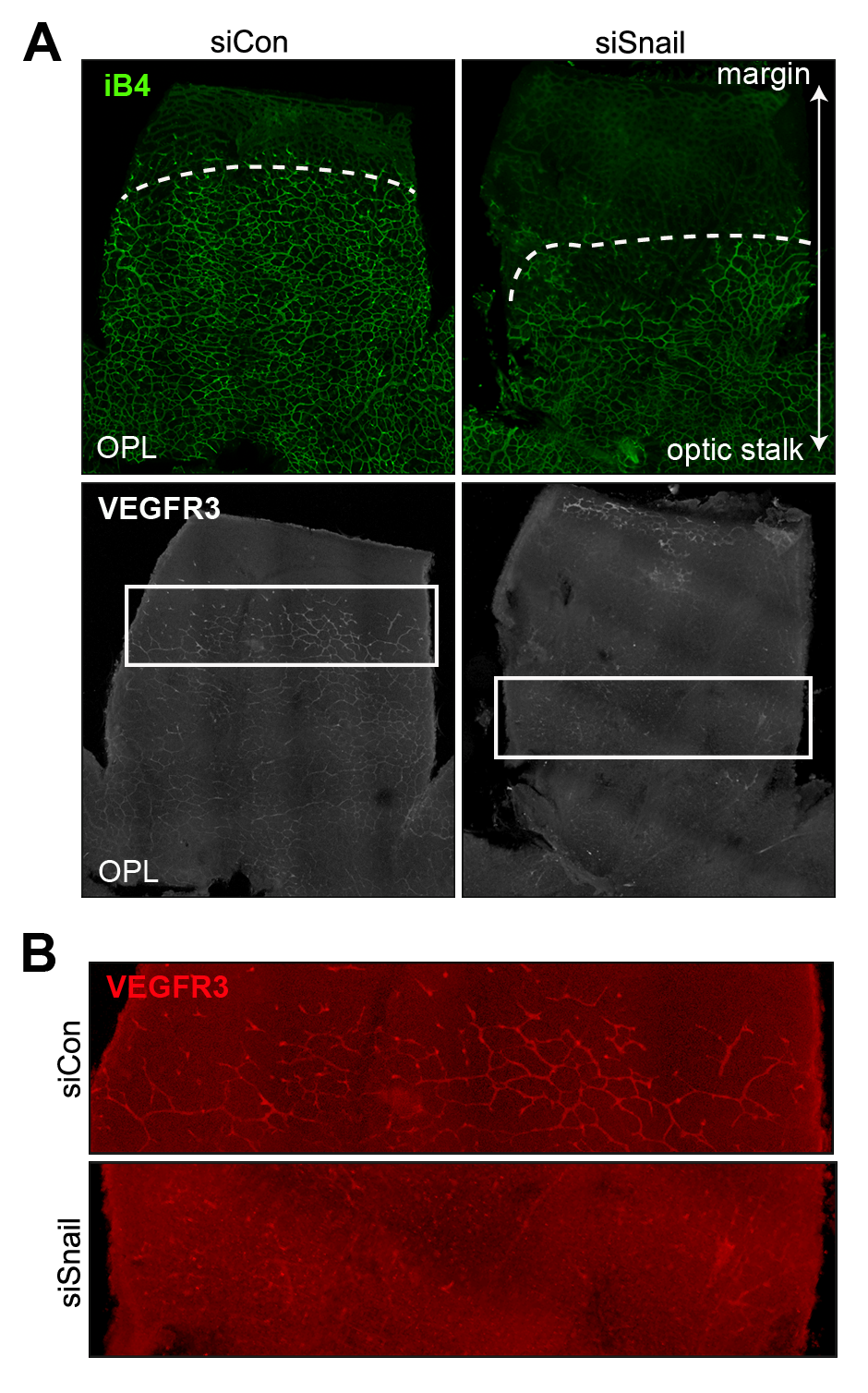

Supplement: S8 Fig — (A) Whole flat-mount immunostainings of iB4 and VEGFR3 in the deep capillary plexus in stable siCon- or siSnail-injected mice. This figure is related to Fig 6C. Stable siSnail-transfected mice exhibited faint expression of VEGFR3 (white) as well as a defect in the deep capillary plexus (iB4, green). (B) Whole flat-mount immunostainings of VEGFR3 were enlarged from white box in A. (TIF) [file pgen.1005324.s008.tif]

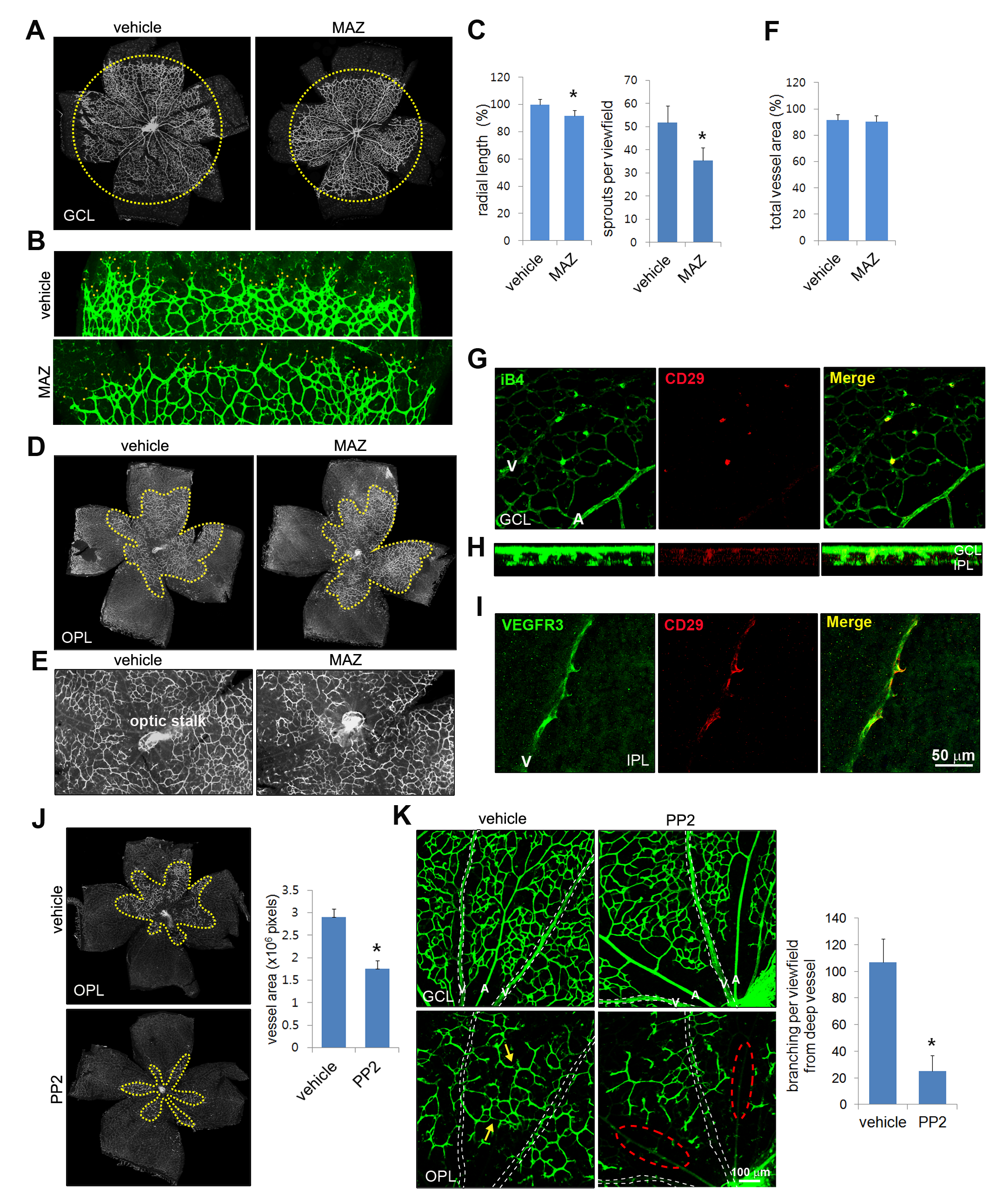

Supplement: S9 Fig — (A-C) Whole flat-mount immunostaining of iB4 in the superficial plexus of retinas after consecutive, intraperitoneal injections of MAZ51 at P4 and P5. MAZ51 efficiently attenuated the superficial vasculature (A) and sprouts at the vascular front (B). Yellow dots in B indicate sprouts. *, p<0.01. Quantification of radial length and sprouts from the retinas shown in A and B (C). MAZ, MAZ51. (D-F) Whole flat-mount immunostaining of iB4 in the deep capillary plexus of retinas after consecutive, intraperitoneal injections of MAZ51 from P7 to P10. The yellow broken-lined circle indicates the vascular area at P11 (D). The vasculature around the optic stalk that enlarged from D is shown (E). The total vessel area was quantified (F). (G) Whole flat-mount immunostaining of CD29 (red) in retinal vessels at P8. CD29 immunoreactivity was observed in vessels that sprouted from venous vessels. (H) Z-stack analysis showing CD29 immunoreactivity (red) in vertical sprouting vessels from the GCL to the IPL at P8. (I) Whole flat-mount immunostaining of CD29 combined with VEGFR3 immunoreactivity in vertical sprouts from the vein at P8. (J) Low magnification of whole flat-mount immunostaining of iB4 in the deep capillary plexus after consecutive, intraperitoneal injections of PP2 from P7 to P9. The yellow broken-lined circle indicates the vascular area at P10 (left). Quantification of vessel area in the deep plexus (right). *, p<0.01. (K) High magnification of whole flat-mount immunostaining of iB4 in the superficial plexus (GCL) and deep plexus (OPL) at P10. Confocal images were taken in the superficial region and the deep plexus by moving the microscopic focus down, along with z-stacks. White-broken lines indicate the position of the vein in the superficial plexus. Red-broken circles indicate vessel-free regions (left). Yellow arrows indicate interconnected vessels by sprouting (left). Quantification of branching points in the deep plexus (right). *, p<0.01. (TIF) [file pgen.1005324.s009.tif]
